# Part I. Spirit of English Periodical Literature

**Published:** 1833-10-01

**Authors:** 


### Periscope;

OR,

### CIRCUMSPECTIVE REVIEW.

"Ore trahit quodcunque potest, atque addit acervo."

### I.

### Spirit of the English Periodicals, and Notices of English Medical Literature.

### I. Our Art in Turkey.

Recent events have drawn the attention of Europe to the state of Turkey, that huge anomaly of modern times, an uncivilized and unchristian empire, preserved by the jealousies of civilization and of Christians. It is more than probable that few years will elapse ere the descendants of Omar are driven from Europe, and we watch their struggles now with feelings akin to those that we experience when gazing on a dying man. Our business is not with political considerations, and we pass to a subject more immediately interesting to ourselves, the condition of our profession in the land of the Turk. It has been said, with reason, that the female sex assume a more commanding station with the progress of civilization; drudges in the Wigwam, they expand into goddesses in the Café. It seems to be otherwise with doctors. In the deserts of Africa, the steppes of the Caucasus, and the plains of Hindostan, the physician is received as a sacred character, his person is secure, his unbelief is forgotten, and barbarous rapine and brutal fanaticism alike cower down before the assuager of human woe.

A very accurate and consequently a very interesting account of the state of medical art and its professors in Turkey has lately been published in Germany by Dr. Oppenheim, and is brought before the English reader by Dr. Robert Graves. This gentleman, in contributing to the Dublin Journal notices of the more valuable modern German works, is doing a service to the profession that we feel great pleasure in acknowledging. From this gentleman's paper in the last number of our excellent Irish contemporary,* we shall extract such passages as we think more particularly amusing or instructive, referring our readers for further details to our contemporary himself. It is scarcely necessary to observe that Dr. Oppenheim possessed great and peculiar opportunities for obtaining the information that he has now made public.

1. *Physicians in Turkey.* The means of making a good physician vary in different countries. In Turkey they are formed as law-givers are with us, hereditarily. In America and France and elsewhere, a prescribed course of medical study, followed by prescribed examinations, constitute the doctor. In England the neophyte swears to the thirty-nine articles, and ruins his health and his morals on the banks of a lethargic stream, where no medicine is taught—and he too becomes a doctor. Thus we see that customs differ. But our present business is with the hereditary physicians of Turkey.

"The father of medicine in Turkey was an Arabian, named Lochmann, appointed in the seventeenth century by Mahommed, to discharge the sacred functions of physician. The miracles performed by Lochmann were nume-

* No. IX. July 1833.

rous, and tradition has recorded them in glowing colours: he was a wandering dervise, and taught his art to the brethren of his order, who, retaining to this day the precious secrets he revealed, continue by birth-right the practitioners of Turkey.* As might be expected, this religious order of physicians are greater proficients in superstition than in practical medicine, and except being acquainted with the virtues of a few plants, they absolutely know nothing. It is true, indeed, that they attempt to acquire confidence by appealing to supernatural agency, divination, astrology, talismans, and cabalistic figures.

Sometimes they attribute the origin of disease to the special wrath of God, in others to the interference of devils, but never perform the ceremony of deprecation or exorcism, without a multiplicity of rites and sufficient pay. Where money is given in the expected quantity, their prayers are endless, their beads are told *ad infinitum,* picked sentences of the Koran are sewn together and given to the patient to swallow; or when a fluid menstruum is preferred, the holy words are written with chalk upon a piece of board, this is washed, and the water with which the ablution is performed, forms a draught potent in proportion to its impurity. Amulets, however, form the favourite charm of the Turks, and over the whole of the east, Mahammedans,† Jews, and Christians, appeal to their protection, when threatened or overtaken by misfortune. Hence, few die without wearing two or three amulets, to whose safe guardianship they had intrusted their lives. They generally consist of a scrap of paper, containing a sentence from the Koran or Bible, embellished with cabalistic figures, and folded in a triangular shape, enclosed carefully in a little bag, and worn next the skin, either by means of a string hanging from the neck, or by being stitched inside the turban. Some amulets, supposed to possess a spell capable of protecting from ball and dagger, are sold at an enormous price."

We think that the Hohenlohës, Irvings, and St. John Longs, have something yet to learn. They have not made their patients swallow their prescriptions. The chalk-mixture is an excellent idea, and must not be confined to Musselmen. The unction with which devils are cast out being regulated by the quantum of the pay, is not without parallel in Europe. They who smile at the fanatic Turk, beguiled by amulets and snatches from the Koran, look complacently enough at the Christian fools, Lords, Baronets, and Countesses, who depose that quicksilver is extracted from their heads, gout from their feet, and water from their brains, at the bidding of a wretched painter. Nay, what is the religious charm held sacred by a people whose existence is the mere consequence of religious fanaticism; what is this, we say, when compared with the mummery of a magnetising needle amidst the most civilized people of the world? And who are they that will sneer at the Mahometan duped by the faith of his fathers, and read without a shrug a medical periodical gravely descanting on the effects of a magnet. Well has Lord Bacon remarked that, "in all times, in the opinion of the multitude, witches and old women, and impostors, have had a competition with physicians,"* and well did the ancient theogonists feign that Æsculapius and Circe were both children of the sun, that sun which Hamlet says breeds maggots.

The Turks, humbugged as they are, yet possess the sense, which some Europeans seem to want, of appreciating the effects of education and study. Accordingly a foreign physician, particularly a Frank, is highly valued, and greatly followed. This, in fact, has led to an abuse which a knowledge of the

* The Turks, with a happy knack of distorting Frankish names, have confounded Hoffman with Lochmann.—Thus Hoffman's liquor they call Lochmann-Rouch.

† The name of the prophet is pronounced *Mahammed.*

* Essay on the Advancement of Learning.

Frank character might easily lead one to suspect.

"The encouragement thus given to foreign practitioners, has generated the greatest abuses; for as there are no means of ascertaining the acquirements of strangers, many, induced by sordid views, embark on a system of barefaced quackery, and thus persons who have followed other employments at home, are suddenly physicians in Turkey. Dr. Oppenheim was invited to attend a consultation with an eminent French physician at Smyrna, who candidly told him, that the only preparation he had for the profession was, service in the army as drum-major! Among the staff-surgeons of the Turkish army, was a Maltese, who had been a letter-carrier at Corfu, and an Italian captain of a merchant vessel, who had been shipwrecked on the coast of Asia Minor. A Genoese gentleman, implicated in the late revolutionary attempts in Piedmont, and who had long served in the army, applied to Dr. Oppenheim, who gave him sixteen recipes, by means of which he was set up in the world, being soon afterwards appointed physician to the Governor of Jambul! Nothing can exceed the heterogeneous materials of which the mass of practitioners is composed; foreigners from all countries, and of all trades, but chiefly Greeks, Jews, and Armenians, the religious orders of all the different forms of worship that are professed in Turkey, besides gipsies, barbers, and old women. Of the foreigners, some are well educated, and a few whose names Dr. O. mentions, are excellent surgeons and experienced physicians, but such are "few and far between." It is a pity, that the state of medicine is so low in a country, where the inhabitants esteem so highly the medical art, and where all are inclined to respect physicians; by the Turks, a skilful physician is almost ranked as a saint, and the appellation "Hekim" is the surest protection against either religious or political persecution. In the last campaign against the Russians, often, says Dr. O., was the uplifted sword of the half-barbarous Turk, arrested on the cry of "Hekim" being uttered by his vanquished foe. The modern Greeks give the title of Excellency to the physician, and old Homer estimated the value of a good surgeon and physician very precisely, in saying, that he was worth half a dozen colonels!" *

There is a particular district of Greece, Sagor in the Paschalick of Janina, where the profession of medicine is the chief occupation of the inhabitants, whose right to practise is hereditary, and whose knowledge consists in recipes handed down from generation to generation. From three or four villages in this district, swarms of itinerant physicians annually spread themselves over the whole of Turkey. They are ignorant enough, as may readily be imagined. Jewish physicians also abound, and are fully as ignorant as they. They are medical pedlars, carrying wallets, and uttering in every public place the shrill cry of "ei hekim, ei hekim," *(a physician, a physician.)* When consulted, they feel the pulse, roar out—"bilirim senin hastalik," (I know thy disease) open their wallets, give a pill or a powder, and receive the thanks of the patient, with a fee of two or three half farthings. On this Dr. Graves or Dr. Oppenheim, we know not which, indulges in a few very ominous bodings.

"Knowledge came from the East, it has travelled slowly to be sure, but here it has arrived at last, and lo! our fees, formerly paid in gold, are *changed* into silver, and undergoing the rapid process of depreciation, the distant tinkling of brass may be heard even now by the ear, practised in the sounds of coming events."

Such a "depreciation of the currency" would be melancholy indeed, but let us hope that the day is yet distant when the fashionable doctor will, after a hard day's work, pull out his coppers, and murmur at the illiberality of Lord E. in not inclosing the customary farthing.

---

* "It is difficult to assign their proper rank to many of the chiefs and minor heroes of the Iliad. In calling them colonels, I mean no offence to the dead."

In Turkey every thing is learnt by the pulse, and by the pulse every thing is determined. In this country there have been "Water Doctors," who were paid, and who prognosticated, and prescribed by the chamber-pot. If Sterne be in the right when he declares that "there are worse occupations in the world than feeling a lady's pulse," the Ottomans have the advantage of us. With them a physician is made or marred by his dexterity in applying his fore-finger to the radial artery. The governor of Adrianople, Halisch Pacha, once visited the tent of the Russian General, Paulin, where Dr. Oppenheim and two other physicians were attending at levee. Each of the three successively was presented to the Pascha, who made them feel his pulse; and when the ceremony was over, he immediately declared, that one of them was imcomparably a better physician than the others, for, said this wise Pascha, he felt my pulse much better! With us, a pet doctor gets his reputation the Lord knows how! Whether such things occur in Christendom as bribing ladies' maids, we cannot say, but the Greek physicians fee the servants to inform them of their masters' and mistresses' *motions*, and subsequently declare them to the astonished patient, on examination of the pulse.

When the physician, by means of the pulse, has declared the precise nature of the disease, and the exact moment of its termination, the Musselman requires him to give a certain medicine, to have some particular effect in determining some evacuation, which is to prove critical. No medicine gets the least credit, or in their eyes can be the least effectual, unless it produce sweat, urine, or purging. The Turk is fond of large doses, too, in order to produce more decided crises, and he always prefers medicine in the shape of a draught, or rather drink, (sherbet.) He dislikes emetics, and nothing will induce him to allow the exhibition of an enema. It is quite vain to endeavour to make him alter his diet; of this he cannot conceive the use. In the Month of May, it is not unusual for them to submit to what is termed the spring cure. An active purgative is first taken, and afterwards the expressed juice of various plants, such as taraxacum, various grasses, &c. are taken daily, along with a drink of whey. The most favourite purifier of the blood, however, is *viper broth*. The most esteemed vipers are caught in the neighbourhood of Adrianople, and are sent thence in great numbers to Constantinople, and other parts of the empire. They are kept in wooden vessels, and when wanted for use they are drawn out through the bung-hole. It is needless to remark, that this operation requires much caution and skill, in spite of which, as happened in an instance which Dr. O. himself witnessed, the poor apothecary is sometimes bitten. The bite often, but not always, proves troublesome, or even fatal. When this dangerous article of the materia medica has been safely extracted from the vessel, his head and skin are instantly taken off, and the animal is cut into thin slices, which are boiled with water to make broth. The most effectual of the means employed either for the prevention or cure of diseases by the orientals, is the bath *(Hamam.)* The long continued frictions employed, the stretching, drawing, kneading of the limbs and flesh, the pulling and working of the joints, &c., all tend to exercise a healthful influence; it is astonishing, what a command over the joints an experienced attendant at the baths possesses. He twists them in every direction, and you almost feel, as if he had performed on you a number of successive dislocations and reductions, following each other with surprising rapidity. In chronic diseases of the skin, gout and rheumatism, these baths are invaluable.

The baths are splendid in conception and construction, so cheap as to be accessible to the poorest Turk, and form for the ladies a good equivalent for morning concerts, or morning calls. We may refer the curious to the modest letters of Lady Mary Wortley Montague.

2. *Fees in Turkey*. The sick Turk, like the sick devil, makes promises: the convalescent Turk, like his prototype, breaks them. In consequence of this disposition, the physician is often obliged to draw up a specific contract in writing, and according to a legal form, before he undertakes the treatment of a case or the performance of an operation. The contract is deposited in the hands of a magistrate, who can enforce payment, and whose zeal in the discharge of this duty is quickened by the legal fee of ten per cent., to be deducted from the stipulated sum. It is not very rare, however, for the patient to evade the ends of justice, by paying the magistrate twenty per cent; when this is done, the physician's contract too often turns out to be waste paper. These contracts, however, in general afford the physician tolerable security, and are especially necessary when capital operations are performed, as without them he may lose not merely his fee, but his life, in case his patient dies, for the Turk considers the knife of the surgeon in the light of a weapon wielded by an enemy, and thinks himself called on to avenge the death of a relative after an operation. This is hard enough upon the poor surgeon, who, to avoid more fatal consequences is often obliged to pay blood-money to appease the wrath of relatives. To avoid these consequences, the surgeon and one of the nearest relatives of the patient repair together to the *cadi*, if it be a small, or to the *mufti* if a large town, and obtain from him a *protection* (fetwa,) by which the surgeon is secured against all persecution if the patient dies. Dr. Oppenheim, himself, felt the force of this Turkish antipathy to the performers of unsuccessful operations. After the battle of Monastir, on the 24th August, 1830, he amputated the leg of a wounded Deli:* the Deli died. In a few months, Dr. Oppenheim was sent by the Grand Vizier to inspect recruits at Pristina, and was invited to the house of the *Cadi*. This honest functionary having been assured that the Doctor had operated on Soliman Aga, the deli, said—"you behold here the father of Soliman-Aga, who claims blood-money from you, which money it is most just you should pay him." Dr. Oppenheim, being protected by the Vizier, soon brought the Cadi to reason.

"When a physician has treated a patient who dies of internal disease, he incurs no risk, unless the deceased held some important and lucrative government post; in such cases, the relatives and dependants of the deceased, being deprived by his death of their station and emoluments, are apt to wreak their vengeance on the physician, who, however, generally takes care to be out of the way on such occasions. At other times, medical men are employed to give opinions, concerning not the living, but the dead! This may appear strange, but it is the fact, and it is for such opinions that they are sure to be best paid, for they have it in their power to make what conditions they please with their employers. In Turkey, whenever a governor of a province, or mufti, or any other *employé* of the government dies, the whole of the treasure in his possession immediately finds its way into the coffers of the state, therefore, it becomes an object of paramount importance for the family, to conceal, if possible, the death of their relative, until they have either made off with his money, or what is a safer method of proceeding, until they have used one portion of it to bribe the members of the divan into conniving at their keeping the remainder. The father of the present Pascha of Uskup, it is now ascertained, was buried four years before his death was announced. During the interval, his son had carried on all the public business in the father's name, and the signature of the latter was affixed to all official documents. During this period, medical advice was sought for in all quarters, and eminent physicians were even brought from Constantinople. They were consulted, but for very evident reasons, were never permitted to see their patient, a matter es-

* The Delis form the flower of the Turkish cavalry, and their name means *madman*. They are so called from their frantic impetuosity in battle.

teemed of little consequence in Turkey, provided the state of the pulse is accurately described."

3. *Apothecaries in Turkey.* These require no Act to bother a Turkish Divan, for this simple reason, that there are none. Indeed there are no shops for the sale of medicine, except at Constantinople, and one or two other large towns. Every physician mixes his own medicine.

4. *Poisoning in Turkey.* No restraints being placed upon the sale, poisoning both by accident and intention is too common. Sometimes medicine is weighed in a scale still soiled with corrosive sublimate or arsenic. Often the ignorant practitioners prescribe powerful medicines in poisonous doses. The writhings of the patients are then interpreted as symptoms of being possessed, and forthwith the Turkish dervise and the Christian Priest are in requisition, and proceed simultaneously with their different forms of exorcism. The precaution of having recourse to the rites of two different religions, is taken to avoid the possibility of mistake or failure, for, say they, we cannot, *à priori*, tell whether our friend is possessed by a Mahammedan or by a Christian devil.

Poisoning by design is still more frequent, and the physician too often the venal instrument of murder. The Turk thinks it no sin to poison an enemy, and for two reasons:—If you do not poison him, he will poison you; and besides, you cannot poison him if he is not fated so to perish. And this is the "brutal and bloody" Empire, that christian jealousies allow to pollute the fairest portion of Europe. The following and concluding extract will put this dreadful laxity in moral principle in a striking point of view.

"Melancholy, as it is," says Dr. Oppenheim, "to witness such mischievous misinterpretation of a Mohammedan dogma, it is still more melancholy to see persons who profess Christianity engaged in the same guilty course, for it cannot be denied that too many of the native Christians of the Greek Church are willing agents upon such occasions. In truth, no honest person ought to engage himself as domestic Physician to any great man in Turkey, for if he be called on to poison, and refuses, it may cost him his life. Of this I myself had a convincing proof. The late campaign of the Turks against the Albanians was brought to a successful conclusion, not by superior courage, numbers, or discipline, but by craft and treachery. Two of the most powerful foes of the Sultan, Whely-bey and Asslan-bey, surnamed the Lion Chief, were invited during a truce, to witness a review of the Turkish regular troops, which to them was a matter of great interest and novelty. The Vizier had it so arranged that they were both shot dead as they were passing in front of one of the battalions. The Vizier's son, Emin, Pascha of Janina, ensnared and despatched some of his most formidable opponents in a nearly similar manner at Janina. One evening at levee, the Grand Vizier made a sign for me to remain, and when all the courtiers had left the room, he ordered in coffee, pipes, and a chess-board, and I then found myself alone in company with a man who expected and received unconditional obedience from every one of his attendants, and at whose nod more than one hundred thousand heads had fallen. Having signified that I should be seated on the divan, he smoked, but according to etiquette, I left my pipe untouched; and when we had made a few moves at chess, he raised his head, looked fixedly into my eyes, and said 'Hekim-Baschi, I have enemies, you can and will assist me!' He then made the sign for me to retire, which of course precluded the possibility of my replying. I made my obeisance, and rode home greatly agitated and alarmed, for the meaning of the Vizier's words was but too intelligible. At that time I was attending two Albanian Chiefs of note, who were afraid to trust themselves to the care of the Vizier's physician, and who had applied to me as an officer of the staff for advice. The Vizier was aware of this, and wished me to despatch my two patients. I revolved in my mind the difficulties of my situation, and saw no other method of escaping than by making large pecuniary sacrifices, in the way of bribe, to the Vizier's avaricious *Seraff*, (Pay-master,) and his *Grammatiko*, (Secretary.) In the mean time I feigned sickness, and remained at home. Twelve days had elapsed since my interview with the Vizier, and nothing remarkable had occurred. On the morning of the thirteenth day, my servant brought in my pipe and coffee as usual; I had nearly finished the cup, when I perceived an unpleasant taste, which excited my suspicion; I immediately took an emetic, and hurrying to the apothecary of the forces, he immediately recognized in the cup nearly two drachms of corrosive sublimate, upon which I swallowed the whites of several eggs, and experienced no further bad effects. Though the favour I enjoyed at Court, and the prominent station to which I had been advanced in the medical department of the army, had made me an object of envy to many, each of whom might wish to see me removed, yet it was but too evident, that the blow aimed at my life had descended from a higher quarter, and, accordingly, I used every exertion to obtain a passport *(buerouldi,)* and, at last succeeding, hastily quitted Turkey."

When any one in Turkey has become remarkable for power or wealth, it is usual to observe—" he will probably soon die of poison." Hence the rich eagerly cultivate the friendship of every newly-arrived physician, particularly of a Frank, lest he be employed to poison them. The sick Turk makes his physician or his slave take part of all his medicine, and the miserable menial vomited and purged for his more miserable master, has good reason to pray for a speedy termination of the malady. When a bottle of physic is opened, and the dose measured out, it is again immediately sealed up with the master's private seal, to prevent the introduction of any poison.

It requires no spirit of prophecy to foretel, that such a people cannot long retain even the most petty empire in Europe. National crimes are national weakness, and when the fruit is rotten at the core, the feeblest hand can crush it.

---

### II. Dr. Graves on the Treatment of various Diseases.

In our last number, we had the pleasure of noticing a communication of this able physician, and we took the liberty of offering some observations on Morbid Anatomy, and on rational and empirical experiments. We lately met with some remarks very pertinent to the subject, and those from no less an authority than the great Lord Bacon. That extraordinary man has pointed out the sins of physicians, whether of omission or of commission, with admirable precision and felicity. He said well, that medicine is a science which hath been more professed than laboured, and yet more laboured than advanced, the labour having been rather in circle than progression. But it is particularly on the subject of treatment that we would beg to introduce Lord Bacon's observations.

In the consideration of the cures of diseases, I find a deficience in the receipts of propriety, respecting the particular cures of diseases; for the physicians have frustrated the fruit of tradition and experience by their magistralities, in adding, and taking out, and changing *quid pro quo*, in their receipts, at their pleasures, commanding so over the medicine, as the medicine cannot command over the disease; for except it be treacle and Mithridatum, and of late diascordium, and a few more, they tie themselves to no receipts severely and religiously: for as to the confections of sale which are in the shops, they are for readiness, and not for propriety; for they are upon general intentions of purging, opening, comforting, altering, and not much appropriated to particular diseases; and this is the cause why empirics and old women are more happy many times in their cures than learned physicians, because they are more religious in holding their medicines. Therefore here is the deficience which I find, that physicians have not, partly out of their own practice, partly out of the constant probations reported in books, and partly out of the traditions of empirics, set down and delivered over certain experimental medicines for the cure of particular diseases, besides their own conjectural and magistral descriptions. For as they were the men of the best composition in the state of Rome, which either being consuls inclined to the people, or being tribunes inclined to the senate; so in the matter we now handle, they be the best physicians, which, being learned, incline to the traditions of experience, or being empirics, incline to the methods of learning.—*Essay on the Advancement of Human Learning.*

In another place, Lord Bacon points out the futility and vanity of frequent changes in plans and prescriptions, one day's method undoing that of the preceding.

But lest I grow to be more particular than is agreeable, either to my intention or to proportion: I will conclude this part with the note of one deficience more, which seemeth to me of greatest consequence; which is, that the prescripts in use are too compendious to attain their end; for to my understanding, it is a vain and flattering opinion to think any medicine can be so sovereign, or so happy, as that the receipt or use of it can work any great effect upon the body of man: it were a strange speech, which spoken, or spoken oft, should reclaim a man from a vice to which he were by nature subject; it is order, pursuit, sequence, and interchange of application, which is mighty in nature; which although it require more exact knowledge in prescribing, and more precise obedience in observing, yet is recompensed with the magnitude of effects. And although a a man would think by the daily visitations of the physicians, that there were a pursuance in the cure; yet let a man look into their prescripts and ministrations, and he shall find them but inconstancies, and every day's devices without any settled providence or project; not that every scrupulous or superstitious prescript is effectual, no more than every strait way is the way to heaven, but the truth of the direction must precede severity of observance.—*Ibid.*

We have introduced these passages, partly as curious in themselves, and illustrative of the extraordinary attainments and sagacity of the master-mind from whence they sprung, and partly as containing suggestions, which it were well even the present generation of physicians acted on more strictly and more universally than they do. In conducting clinical experiments on the powers of medicines, it is necessary, to make them of value, that the case should be freed from adventitious circumstances, and that the remedy should be as simple and uncomplicated as possible. To shew the necessity of these cautions, we will take a contrary instance. A poor child is admitted into a hospital, where it is well housed, well clothed, and well fed. At the same time, iodine is given for a scrofulous complaint. The child improves. Is the improvement owing to the exhibition of iodine, or to the alteration in temperature, clothing, diet, &c.? It is manifest that the experiment is an inconclusive one. We might cite many instances, differing in the particulars, but similar in the principle, and tending to the same result—an imperfect or a false experience.

There is no doubt that the great improvements that have recently been effected in the practice of medicine have been owing to the cultivation of morbid anatomy. But it must be recollected that there already existed a vast store of empirical experience, which the exact discoveries of morbid anatomy enabled us to apply. This empirical experience was itself the result, in a great degree, of chance, or of ignorant experimenters. The invention, then, of remedies, and the accurate knowledge of disease, are not necessarily connected. Remedies may be numerous, and a disease misunderstood; a disease may be well known, and remedies undiscovered. Circumstances have made the invention and accumulation of remedies precede an exact acquaintance with disease. The latter part of knowledge is of recent date—the former has existed and grown since the æra of the first old woman that culled a simple.

It now becomes the province of scientific men to conduct experiments on rational principles. With the real nature of disease they are now almost as well informed as the investigation of structural changes can render them. It is their part to repeat in a more philosophic manner the experiments of their predecessors. They must not overrate morbid anatomy; it will not of itself teach them how to cure. The vast extant store of materia medica is an instrument in their hands, and means yet untried no doubt exist. Great inventions are generally in their origin mean and fortuitous; philosophy is usually concerned in their application and extension.

We make these remarks, imperfect as they are, and impertinent as to some they may appear, in order to induce physicians to conduct their clinical experiments in a philosophical manner. It is by these that our science may be materially improved, and it is by their combination with the demonstrative element of morbid anatomy, that it may assume something like the aspect of exactness: and this brings us back to Dr. Graves. The first fact related by him is presented as not only remarkable in itself, but as calculated to impress on every practitioner the propriety and necessity of an accurate examination of every case.

*Fracture of a Rib produced by a violent Fit of Coughing.*

On March 24th, 1833, a lady, æt. 47, tall and unusually muscular, consulted Dr. G. on account of pain in her left side. She had no fever, but inspiration was attended with extreme pain, felt in the situation of the left kidney and shooting upwards to the left shoulder; there were also great soreness and tenderness extending in every direction from central portions of the ninth and tenth ribs. The lady said that she had been seized with the severe pain five days previously during a severe fit of coughing, and that she had experienced the sensation of something having snapped. Leeches and a blister had been applied without relief.

Dr. G. was a little puzzled, but subsequently examined the lady in bed. He then found that the central point of tenderness was seated, not between the ribs, but on one of them, either at or very near the junction of the cartilaginous and osseous portions. Pressure here could scarcely be tolerated, and conveyed the sensation of the bone yielding—in fact, of its being broken. The lady observed that such had been her own suspicion, that she had felt as on a previous occasion when she broke her arm, and that she was easier when her stays were on. On applying a compress and roller, the lady immediately experienced relief, and gradually recovered without any other remedy being used.

In this instance there was no evidence of peculiar fragility of the bones, nor was there any disposition to scirrhus. Dr. G. is not aware of any recorded instances of fracture of ribs by muscular exertion. He cites a case of fracture of the sternum by muscular action, related by Dupuytren, and detailed in the April number of this Journal, page 532.

*Suffocative Catarrh.*

"Many have written on the best means of affording relief when the patient seems in danger of being suffocated by the accumulation of fluid secretions in the bronchial tubes. In such cases the secretion, instead of being scanty, is superabundant, and as long as the patient has strength, is easily expectorated. The very abundance of the secretion, however, and the constant necessity of expectoration, interferes with the function of aeration, and at length the sufferer becomes so weak that he coughs up with difficulty the sputa that obstruct the passage of air into the lungs. Each effort to do so fatigues him excessively, and adds to his debility; his countenance becomes more and more suffused and livid; the rattling of mucus is heard within the chest; the perceptive and mental faculties are dull and impaired, and, finally, the patient is suffocated after a painful and protracted struggle. This series of symptoms frequently attends common cold in the chest in those who are debilitated by great age, and is not unusual in younger persons after a severe bronchitis which has lasted until their strength has been broken, and an excessive flux from the mucous membrane of the air-passage has been the consequence of its long continuance. The late epidemic influenza, in consequence of the extreme and immediate debility, and the violent determination to the mucous membrane of the air-passages which it occasioned, was a disease peculiarly well calculated to produce the state of things above described; and, accordingly, it often terminated in suffocation, from the accumulation of mucus in the lungs. This state must be carefully distinguished in practice from the dyspnœa and tightness of chest accompanying a difficult and scanty expectoration, for stimulants are often serviceable in the former, but never in the latter. When the danger is from excess of secretion and accompanying debility, we can only attempt a cure by medicines calculated either to diminish the quantity of fluid to be expectorated, or by means adapted to increase the patient's strength. Practitioners have sought to effect either or both of these objects by various means. Emetics, stimulating expectorants, such as decoction of polygala, with carbonate of ammonia, balsam of copaiba, combinations of antimonials, squills, and ipecacuanha, lac ammoniaci, mistura ferri composita, the frequent change of the patient's position in the bed, the inhalation of various vapours capable of stimulating the respiratory apparatus to renewed action, the application of blisters to the chest and nape of the neck, of the actual cautery along the course of the eighth pair of nerves, the use of wine or punch, have all proved occasionally successful in cases of this nature. Still, however, the instances of failure are so numerous and distressing, that it becomes the duty of every physician to seek for means still more efficacious and certain. Tonics and opium are well known to possess a powerful influence over the secretion of the bronchial tubes, and it has been long observed that when injudiciously exhibited, they often suddenly check expectoration, tighten the chest, and bring on the most formidable dyspnœa. A knowledge of those baneful effects induced me to hope that these medicines might be so managed as to relieve the affection of the chest, in which suffocation is the result of superabundant secretion and debility. As all practical men were agreed that sulphate of quinine and opium, exhibited in the usual way, had failed to produce relief in such cases, I determined to try these medicines in the form of injection. Nor was an opportunity long wanting for the trial, as in the course of a few days I was called by Mr. Wallace, of Townsend-street, to see a tradesman's wife residing at Ringsend, and who was apparently almost moribund in consequence of a violent attack of influenza. The quantity of mucus she had expectorated during the last forty-eight hours was quite enormous, and she had scarcely enjoyed a moment's repose, so perpetually was she urged by the necessity of coughing up this superabundant secretion. Within a few hours her strength had appeared quite exhausted, her countenance became livid, and she lay with the sputa rattling in the air-passages, until the imminent danger of suffocation roused her from a state of apparent torpor to make an occasional effort to expectorate. To add to her sufferings, she had been affected with a diarrhœa during the preceding night, and her stomach was so weak that she had vomited once or twice during the morning. In this complicated and embarrassing combination of symptoms, it occurred to me, that besides applying a large blister to the nape of the neck and between the scapulæ, it would be well to use an injection of sulphate of quinine and opium for the purpose of checking the expectoration, and accordingly I directed the immediate use of an enema consisting of three ounces of solution of starch, ten grains of sulphate of quinine, and twenty drops of laudanum. I must confess my anticipations of benefit from this expedient were so slight, that I did not venture to call on our patient next day without previously ascertaining whether she was still alive, for in truth I did not expect that she could have survived the night. I was most agreeably surprised to find that she had slept very much since the administration of the lavement, and that the expectoration had remarkably diminished, while her breathing had become free, and the oppression of the chest less. Her strength had improved, and the lividity of her face disappeared; and, on the whole, her state, although not free from danger, was most satisfactory. The usual method of treating such cases was sufficient to complete her recovery."

Dr. Graves alludes to two other cases, each severe, in which the same means were productive of a similarly fortunate result. He remarks that emetics are sometimes remarkably effectual, but that they often fail, the stomach not being affected by them. In such cases Dr. G. warns the practitioner against exhibiting one emetic after the other in the vain hope of producing vomiting. He recommends, in such circumstances, a combination which he has lately used with considerable success, five grains of mustard-seed in powder and one of ipecacuanha, exhibited every hour or every second hour, according to the urgency of the case. When it is not desirable to excite vomiting the dose should not be repeated at too short intervals, but generally in bad cases there is no such danger. A patient in the Meath Hospital lately took, with temporary benefit and without nausea, fifteen doses in twenty-four hours. Dr. Graves recommends these remedies as addition to our stock in hand, fully conscious that all will occasionally be found unavailing.

*Mortification of the Liver.*

This is so rare that an unequivocal case is a valuable acquisition to pathology.

*Case.* Michael Brien, æt. 50, admitted into Sir Patrick Dun's Hospital, March 4th, 1833.

Ascites—œdema of the legs and feet; jaundice; on examination the liver felt forming an extensive tumour in the right and left hypochondria and epigastrium, hard, and painful when pressed—incapability of lying on the left side; pulse frequent, small, hard—tongue foul—great debility.

Has been subject to pain in the side, cramp and wind in the stomach, and some swelling of the abdomen for six months. The present attack commenced three weeks ago.

From the day of his admission till that of his death, on the 15th March, he progressively grew weaker, and suffered much from tympanitic distention of the bowels. During the whole of the 14th he complained of excessive abdominal pain and tenderness, and in the evening he suddenly began to vomit an extremely fetid fluid, mixed with a dark grey substance. The vomiting recurred at intervals till 5 o'clock of the following morning when he died, without suffering much pain immediately previous to his death.

*Dissection.* On opening the abdomen, about a gallon of serous fluid, deeply tinged with bile, flowed out; the liver was greatly enlarged, extending below the umbilicus, and part of its surface was covered with recently effused lymph. Large, white, solid formations, resembling cartilage, studded its surface, and were found in its interior. They were cupped or concave on the surface, and homogeneous and consistent in their texture. Between them, the texture of the liver was every where healthy. In the inferior portion of the left lobe there was an excavation larger than a man's fist, and half filled with a dark grey slough, of an extremely offensive smell, and precisely similar to the substance he had vomited. This slough was very dry, its fluids having probably been drained off by the large opening which formed a communication between the excavation in the liver and the stomach; the sloughy appearance extended to that part of the pancreas which lay in contact with the stomach, and another perforation of the latter had been formed in this place. An evident line of demarcation existed round the hepatic excavation, resembling the line of separation in external parts.

It appears to us that this is not a satisfactory instance of mortification of the liver. The viscus was studded with the tubercular formations (tubercles of medullary sarcoma,) and one of these would seem to have sloughed, as morbid formations in all tissues not unfrequently do.

*Purpura Hæmorrhagica.*

"This disease is by no means unfrequent among the children of tradesmen and petty shop-keepers in Dublin, and its cause may often be traced solely to unwholesome diet, in which too much salt is habitually used. Practitioners are sometimes deceived, for on questioning the patient, he answers that his parent's circumstances are comfortable, that he has plenty to eat, and not unfrequently even gets meat for dinner. On a more accurate examination, however, it appears that the breakfast consists of bread, salt butter, and tea with very little milk; that herrings or other salted fish are often used at dinner; that bacon is also a favourite article of food, and that the supper consists of the same materials as breakfast. At dinner, too, salt, or salt butter are used to give a relish to the potatoes; and it is only occasionally that these persons enjoy the luxury of fresh meat or vegetables, the earnings which ought to be applied to the purchase of such articles being too often expended in dram drinking. This species of diet, together with confinement and unwholesome air, renders them peculiarly liable to *purpura hæmorrhagica,* in a form which merits the name of land scurvy. My only reason for mentioning this circumstance, is, because I have seen the true cause of the disease occasionally overlooked in our hospitals, and patients subjected to different, and sometimes rather violent, plans of treatment, when all that was necessary for their cure was a nutritious dinner of fresh meat and vegetables, with milk instead of tea for breakfast. Under the influence of this change of diet, it is curious how rapidly the spots of purpura fade away, and how quickly all tendency to hæmorrhage ceases. In the meantime the pulse, which had been so frequent as to induce an inexperienced observer to adopt the idea that the disease is of a febrile nature, diminishes daily in frequency, and this under the influence of nutritious diet, a circumstance well worthy of attention. I think that the cure is accelerated by the internal exhibition of citric acid, to the extent of half a drachm or more daily properly diluted and sweetened."

We feel the more pleasure in directing the attention of professional men to these few remarks on purpura, and facts connected with the disease, because we fear that the practice of blood-letting in cases of it, has too great a number of admirers. We made some observations on this subject in our last number, and to it we beg to refer our readers.

*Effects of Jaundice upon Vision.*

That "all seems yellow to the jaundiced eye," is known to be a poetical expression of a vulgar fiction. But Dr. Graves has lately met with three cases in which the vision was affected by the discoloration of the organ. Our able author enters into many considerations to show why vision should and why it should not be altered. Into these we need not enter. We may simply observe that, supposing the cornea and the humors to be jaundiced, there is no good reason why all objects should acquire a yellow tint. If we look through a portion of yellow glass not deeply dyed (and the cornea and humors have seldom a deep tint) we retain the faculty of appreciating varieties of colour, though yellow is of course made to enter into their composition. There are few patients who, if accurately questioned, are not sensible of such an alteration to a greater or less degree. This, at least, has been the case with those whom we have interrogated.

*Emmenagogues.*

Dr. Graves made some some remarks on a former occasion on emmenagogues.* He omitted to make mention of one remedy, and that a good one, a small blister applied to the inside of the thigh, near the pudendum, a day or

---

* See our last number.—*Ed.*

two before the expected period. In a recent and very obstinate case this proved successful.

*State of the Pulse in Hydrocephalus.*

Acute hydrocephalus has been divided into three stages, the pulse and other symptoms presenting remarkable alterations in each. Dr. Graves reports a case illustrative of this.

A boy died in Sir P. Dun's Hospital on the 15th day of the disease. The disease commenced with violent febrile symptoms, when the pulse was, no doubt, frequent. He was admitted on the 8th day. The pulse was on that day 58 ; ninth day, 68 ; tenth day, 80 ; eleventh day, 92 ; twelfth day, 104 ; thirteenth day, 100 ; fourteenth day, 135 ; fifteenth day, 168.

The following is the relation which the symptoms bore to the pulse.

*First.* When the pulse was only 58, it was irregular, and continued so for two days. After that, the irregularity began to diminish daily, and on the last day all irregularity had disappeared.

*Secondly.* When the pulse was 58, it was soft; when 168, it had become sharp ; at the former period it was weak, at the latter of good strength.

*Thirdly.* When the pulse was at its slowest, the pupils were natural and contracted as usual. As the pulse rose, the pupils became more insensible to light. Yet though insensible to ordinary light, the pupils contracted on the day of the boy's death, when exposed to the light of a candle held close to the eye. On the 14th day, when the pulse was 135, the pupil of the left eye was more dilated than the right.

*Fourthly.* The convulsive fits, a prominent feature in the third stage of hydrocephalus, did not take place till the 12th day, when the pulse was 104. The fits rather affected the right side of the body, the left eye was distorted and squinted permanently, and the mouth was frequently drawn to the right side during the paroxysms.

Let us now look at the morbid changes.

*Dissection,* 16 *hours after death.*—"On raising the calvarium, the vessels on the dura mater were found extremely turgid. On raising this membrane, the pia mater was seen greatly congested; some effusion, tinged with blood, on the surface of the *right* hemisphere; *much greater* on the *left,* particularly towards the posterior part. The arachnoid presented a few slightly opaque spots on the upper surface of both hemispheres, and one continuous, thickened, and opaque patch on the inferior surface of the anterior lobes. On removing the cerebrum from the skull, about four ounces of serous fluid were observed in the base of the skull, and occupying the space around the medulla oblongata. The substance of the brain in general was rather softer than natural, but not more vascular. The lateral ventricles contained about six drachms of sanguineous serum; their lining membrane was increased in thickness and rendered semi-opaque. The *septum lucidum* had been entirely converted in a white shreddy mass which fell from its position, leaving an opening between the lateral ventricles of the size it had presented in the healthy state. This mass lay in a semi-fluid state in the anterior and lower part of the third ventricle. The *fornix* presented a remarkably soft state of its entire lower surface, *more especially at the right side,* where the softening extended through its entire thickness, and a deficiency of its margin, about three lines long, and one line in breadth, existed; the white substance which had *fallen out of* this deficiency lay in a pulpy mass on the plexus choroides; the surface of the velum interpositum was dotted with white matter fallen from the formix. The commisura mollis had nearly disappeared; the superior surface of the optic thalami was soft, but this state was there extremely superficial; *their inner* surface, to the depth of *a line,* was converted into a *blueish white* jelly. The plexus choroides was turgid, and closely studded with very minute vesicles. The white matter which has been noticed as having fallen was pulpy, and resembled white fibres connected by an extremely thin jelly."

Dr. Graves makes a few judicious observations on the case, which we notice in order to point out the concurrence between symptoms and morbid changes. As Dr. G. remarks, two series of morbid alterations were found, one consisting of meningeal effusion and opacity, seen also in the arachnoid of the ventricles, a comparatively early affection : the other more recent, consisting of changes in the brain, the result, apparently, of a degree of inflammation of the cerebral substance. In the early stage we had fever, the expression of the meningeal inflammation, followed by the stupor, dilatation of the pupils, &c. the expression of the consequent effusion. In the last stage, there was again fever, with partial paralysis and convulsions, the expression, not of mere pressure on the brain, but of the cerebral inflammation and irritation. A case of this sort, well observed and reasoned on, will afford more practical hints to a philosophical mind than we have space to dwell on, or mere routinists possibly may comprehend.

---

### III. DANGER OF EXAMINING FEMALES.

A pregnant instance of the risks incurred by medical men, in the incautious examination of female patients, has recently occurred at Liverpool, and given rise to a sort of judicial investigation.

It appears that a young woman, æt. 21, who had been in a bad state of health for two years, applied to Dr. Baird, physician to the Liverpool Infirmary, and, as we are informed, a practitioner of twenty years' standing, in the early part of last March. She was then suffering from debility, dyspnœa, constant thirst, occasional severe headaches, painful and irregular menstruation, frequent desire to make water, the legs and feet œdematous and painful, the veins of the right leg slightly varicose, and pains in the hip and thigh. Active remedies were several times prescribed, but no material benefit was obtained. About the end of March, she first shewed the Doctor her heel, on which was a piece of thickened cuticle, resembling a corn. At the next visit, the thigh having become swollen, in addition to the former symptoms, Dr. Baird proposed a private examination, to which the girl consented. The inguinal glands were found enlarged, but no uterine disease was discovered. At the present time the patient is nearly restored to health, after a continued course of alterative medicines, with digitalis, squills, and quinine.

Such is the statement of the case furnished by Dr. Baird. But this was not all. The girl, it would appear, related the circumstance of the examination to one of those elderly females who are found in or about most communities, and who generally prove a great blessing or a great curse to the generation of doctors. In the present instance, the veteran spinster excited some little hubbub, and the family complained of the indecency of the proceeding.

A gentleman, a reverend one, getting scent of this, complained to the Committee of the Liverpool Infirmary of the atrocious conduct of its physician, in examining the young woman. A junto of three, of which the reverend accuser was one, was delegated to inquire into the affair, and this impartial and well-qualified tribunal would seem to have "pronounced definitively against their officer," and even requested him to resign.

We will not stop to comment on the absurdity of much of the proceeding. The patient was not an infirmary patient, and, consequently, the case was not one for the jurisdiction of the reverend bench. If a man is condemned by the laws of his country for crime, or for any serious moral offence, the governors of an institution to which he may belong, are justified in taking steps to remove from among them one so unfitted for an honourable station. But that the rumour of a private faux pas, the mere suspicion of something wrong, should justify a public board in proceeding to hold a court martial on an officer of the establishment is a measure so unjust, so inquisitorial, so monstrous in every point of view, that all of right feeling must turn from it with disgust. All who know the nature of medical reputation are aware that, to breathe on it the breath of suspicion, is often to taint it irremediably. The prejudices of mankind are concerned, and with prejudices it is vain to reason. What, then, must be the responsibility of those who could lightly lacerate the feelings, and perchance blast the fortunes of a medical man, in subjecting him to an inquest of such a character on such an occasion! We do not envy them, and least of all that clerical inquisitor, at once the accuser and the judge.

Bad as the business is, it is calculated to teach an useful lesson. A female should never be examined unless another female be present. This precaution should never be overlooked by those who wish to escape the imputations to which Dr. Baird has been subjected.

---

### IV. Yellow Vision in Jaundice.

In our present Number will be seen some remarks on this subject by Dr. Graves. Dr. Elliotson, in his medical lecture, reported in our valuable contemporary, the Medical Gazette for July 13th, has some observations on it to which we are tempted to refer.

Dr. Elliotson states that Dr. Pemberton saw two instances of yellow vision, and that in both the jaundice was not very intense. Hoffman also saw two patients, who declared that all they saw seemed yellow. Dr. Elliotson has had two patients who have made the same statement, and, before citing Dr. E.'s account of them, we may premise that he attributes the yellowness of vision to the circumstance of inflammation existing in the cornea, and vessels carrying yellow serum colouring the medium. Whether this explanation be correct, we leave to observation to determine; but the following are the grounds for it.

"In July, 1826, I had a case of icterus in the hospital, where there was albugo of each eye, particularly of the left; and into this eye two large red vessels ran, and with it the patient saw yellow; but the right eye, which had no inflammation before the cornea, into which no large vessels were running, saw things in their natural colour. In 1827 I had another hospital patient who saw yellow with both eyes, and in him the conjunctiva immediately around the cornea, quite at the edge of the orbit, was greatly inflamed. I saw this morning a patient at St. Thomas's labouring under jaundice, who says that at the beginning of the disease he saw yellow. He does not know whether his eyes have been inflamed, but it is a fact that there are several pretty large vessels running not quite to the cornea, but pretty close to it. When patients see yellow, it is from the serum of the blood being conveyed before the pupil, through the cornea. It must be accounted for in that way. In the second case which I met with of this occurrence, I was prepared for inflammation of the eye by having noticed what I did in the first case. I looked carefully at the man's eye the instant he told me that he saw yellow, and I found, as I expected, that it was in a state of inflammation."

The fact appears to be, that sufficient attention has not yet been paid to the subject to enable us to determine the real and efficient cause of yellow vision, when it exists.

---

### V. Diseases of the Eye in Cholera or after it.*

Mr. Middlemore noticed several cases of affections of the eye as a consequence of cholera, when it prevailed last year around Birmingham. The affections were chiefly amaurosis—extensive lymphatic, or puro-lymphatic deposition between the lamellæ of the cornea—ulceration or sloughing of the cornea—and suppuration of the eye-ball. In nearly every instance the disease occurred as the symptoms of cholera were subsiding.

*Amaurosis.* Sometimes this was complete, and often attended with scintil-

---

* Med. Gaz. No. 41, for July, 1833.

lations or painfully vivid corruscations; sometimes it was partial and accompanied with muscæ volitantes. The patients had not generally had cholera very severely, but had suffered an unusual degree of pain in the head during its continuance.

*Deposition between the Lamellæ of the Cornea.* This mostly consisted of lymph or pus, or both. Sometimes it was inconsiderable—sometimes so abundant as to occasion sloughing of the cornea, or absorption of its lamellæ.

*Ulceration and Sloughing of the Cornea.* Sometimes this seemed to occur, without having been preceded by any appreciable amount of inflammation.

*Suppuration of the Eye-ball.* In every case the eye had sustained no inflammation adequate to the production of much pain. Uneasiness only occurred when the globe became distended in those instances where the cornea did not ulcerate and slough, and permit the evacuation of the fluids contained in the eye-ball.

We give the foregoing as facts, and they do not seem to us to require or admit of satisfactory comment.

---

### VI. CROTON OIL AS A COUNTER-IRRITANT.

This is recommended by R. Hobson, M. D. Cantab. It is rubbed on the part repeatedly, so as to keep a fresh crop of vesicles continually rising; two or three drops, night and morning, will usually be found sufficient. The person applying the oil should not look at it, while rubbing it, in order to prevent the occurrence of a troublesome swelling of the palpebræ.

A very good means of obtaining a copious vesicular eruption, speedily and powerfully, is inunction with a mixture of the ung. hyd. fort., iodine, and tartarized antimony. It is an excellent application to the scrotum in the decline of "hernia humoralis," to facilitate the absorption of the effused lymph.

Dr. Hutchinson, physician to the General Hospital at Nottingham, has given a minute description of the local effects of frictions with croton oil. He has also related some cases in which the counter-irritation was beneficial. As we may suppose that there is nothing peculiar in the irritation, and may fairly presume that it does no more than equal counter-irritation produced in any other manner, we need not allude to particular cases. The following is Dr. Hutchinson's account of the effects of the friction.

"Six drops of croton oil, when applied to a sound skin, and rubbed in for a period of from eight to twelve minutes, speedily produces a rubescence, to a greater or less extent, depending upon the individual's susceptibility; this gradually increases, until a general, though moderate, tumefaction occurs, apparently affecting parts deeper seated than I have seen occur from the use of any other external irritant. This is succeeded, in a period varying from six to twelve hours, by numerous vesicles, some distinct, others confluent, differing in size and shape; at first containing a merely limpid serum, afterwards a distinct and consistent pus, and terminating in slight scabs. The redness produced is not of a vivid, but of a dull brick-dust hue. These circumstances, though regular in their course, vary much in intensity, according to the parts upon which the oil is applied."—*Lancet, May* 18*th*.

---

### VII. CAROTID ANEURISM, SAID TO BE SUCCESSFULLY TREATED BY OPERATING ON THE DISTAL SIDE OF THE TUMOUR.*

Although the establishment of weekly medical journals has proved, and is likely to continue to prove, of essential benefit to the profession, the advantage is not without alloy. We regret to observe that our contemporaries are too often in the habit of publishing cases still in progress, sometimes from hospitals, sometimes from private practice. We

---

* Lancet, June 29th, 1833.

need not remark on the mischiefs of this practice, for many read the early part of the case who never happen to light on the conclusion, and very erroneous impressions may be thus produced. We have had enough of false facts, without this formidable addition of imperfect ones. The instance before us is in point. Our contemporary, the Lancet, has headed the case as "carotid aneurism *successfully* treated, by placing a ligature on the distal side of the tumour." With what fairness this may be considered a successful case we shall now proceed to shew.

*Case.* The subject was a free black, tall, rather of a spare habit of body, but emaciated and debilitated. He had been for some time afflicted with a left carotid aneurism; had considerable cough, with irritation of the trachea, and frothy mucous expectoration, palpitation of the heart, great anxiety, and a fixed pain in the left temple. The tumour pulsated strongly, was very large, nearly of triangular form, the base occupying the space of two-thirds of the sternal portion of the clavicle, and ascending nearly four inches from the clavicle to the angle of the jaw, so that the volume of the tumour limited exceedingly the space for operating.

With care, and some very trifling difficulty, the artery was tied above, as it would seem, the omo-hyoideus muscle. On the 4th day, the first dressing was removed. The patient "appeared to be doing well," the pain in the temple having subsided immediately after the operation, and the cough, palpitation, and other distressing symptoms gradually giving way. The volume of the tumour was very perceptibly reduced, and the pulsation very indistinct. The operator was Mr. Montgomery, R.N. Surgeon of the Civil Hospital, Mauritius, and he receives much commendation for the manner in which he operated.

The report proceeds no farther than we have done, and we put it to any one of common sense or common candour, whether our contemporary is warranted in styling this a "successful" operation, or whether, in fact, it is desirable to publish cases at such a stage, whether they are styled successful or not. We are sure that science receives no benefit from their publication.

---

### VIII. What Syphilis and Mercury can do.

Those who are anxious to solve this problem may easily do so, by walking into the foul wards of any large hospital, or by visiting a Lock Hospital, or even by looking at a very ungainly wood-cut, *not* after Titian, exhibited in the first page of the Lancet for the 15th June last. The print is from a sketch made by Dr. Weatherill, of Liverpool, a gentleman who has acquired some reputation in connexion with extirpation of the uterus, and who made some remarks on saline injections in cholera, rather characterised by boldness than by charity. But to the point.

We do not altogether admire the spirit in which Dr. Weatherill's narrative is drawn up, nor do we feel inclined to encourage the tone in which a fellow-practitioner is spoken of. If the circumstance of his being a "dispensary doctor" constitutes a crime in Dr. Weatherill's eyes, it does not follow that the gentleman should be publicly scarified on that account, and with all the deference imaginable for Dr. W.'s good nature, we think that the fault might have been stigmatized, without such marked and painful reference to the individual. We are tempted to notice the case, because we think it well calculated to instil an useful lesson, and because the injurious consequences of the injudicious exhibition of mercury cannot be brought too often, or too prominently, under the notice of the profession.

*Case.* The unhappy individual, whose vestige of a physiognomy is consecrated by the pencil of Dr. Weatherill, a widow, now æt. 38, applied with "symptoms of primary lues venerea" at the Liverpool North Dispensary, in the Winter of 1829. She was attended by one of the Surgeons for a period of eight months, and "was several times salivated." The disease "crept on"—secondary symptoms occurred—"mercurial eruptions" appeared—"the throat, gums, mouth, and fauces, nose, eyes, and skin, were each and severally attacked by the combined forces of syphilis and mercury," and the surgeon would seem to have abandoned the case in despair.

The patient was now in a frightful condition—the face was extraordinarily mutilated by sores, the eyes, nose, mouth, &c. presenting no traces of what they should be, the lips completely sealed together, a circular opening only existing in site of the left angle—hearing impaired—and the whole a miserable picture of disease and destruction.

The patient was sent by some benevolent persons into the country, and owing to its influence, combined with tonics, the general health was considerably bettered, and the appearance slightly so. In March last she gave birth in the poor-house to a fine and healthy-looking boy. He died at the end of three weeks in convulsions; at the date of Dr. Weatherill's report, June 6th, she was dying.

Before we make any remarks on this really melancholy case, we must take the liberty of introducing a few remarks from the pen of Dr. Weatherill.

"Before closing my paper upon the case of this poor, unfortunate woman, I shall take the liberty to offer an observation or two on the medical treatment she received while under the professional care of the dispensary surgeon. The patient applied at the dispensary soon after the irruption of the complaint, and though she was attended for a period of eight months, yet the disease was suffered to go on to a most ruinous, fatal extent! In individuals infected with syphilis, and who allow it to commit its depredations on the system and constitution, uncontrolled because unresisted, I readily grant the mischief will soon be extensive and serious; and finally terminate, almost with certainty, in the destruction of life; but when an opposite course is pursued,—the incursions of the enemy anticipated and repulsed at the onset,—there is little or nothing unfavourable to be apprehended; the disease becomes disarmed, and the patient is soon after restored to the wonted health and vigour."

That they who live in glass houses should throw no stones, is a proverb probably familiar to Dr. Weatherill, and we venture to say that, when liberally criticising his neighbour, he little thought he was exposing himself to criticism. Yet we are sure that the remarks of the Doctor and the sketching of the case, display notions with respect to the venereal disease, of no little crudity and incorrectness. Where, for instance, did the Dr. discover that syphilis, if uncontrolled, would finally terminate, almost with certainty, in the destruction of life? Has the Doctor ever heard of the experiments of our army surgeons? If he has, he must have known that his dogma was opposed to facts—if he has not, we would recommend him to peruse them. Under any circumstances he has no right to speak so magisterially.

It cannot be denied that the case before us is a lamentable instance of maltreatment. Dr. Weatherill asserts that the ravages which occurred, were the consequence of the combined action of syphilis and mercury. We venture to say that they were not; we venture to declare that they were the consequence of the highly injudicious use of mercury alone. What the character of the primary symptoms was we are not informed, and we cannot, therefore, speak to the propriety or impropriety of employing mercury in the first instance, but that repeated salivations are most improper is as clear as any fact of the kind can be. It is astonishing how blindly mercury is given and persisted in by the great majority of medical men, even by those who should know better. Not a day elapses without our witnessing instances of this sort of mala praxis, either in public or in private practice. A patient has a sore, and mercury is given to a considerable extent. The throat then becomes ulcerated. Oh! says the surgeon, here is a secondary symptom, and he perseveres in his mercury, or after a slight intermission prescribes another course. A pustule now appears on some part of the skin, it scabs, and an ulcer results. A fresh secondary symptom! says the surgeon, and again is the mercury in requisition, until at length the patient falls into a state of complete cachexia, or happily consults a more judicious medical adviser, who orders mild aperients, sarsaparilla, country air, or some of the many other means of restoring the general health. Unfortunately this is no imaginary portrait. The original has sat for it within this month.

The medical world are impressed with the idea that, cæteris paribus, if a sore or an eruption gets no better, it is an indication for more mercury. Cæteris paribus, it is an indication for precisely the reverse. The golden practical rule in the treatment of the venereal is this—never to put a patient on mercury when there is much inflammatory action—never to suffer the patient's general health to droop, but to support it by moderately good living and tonics—and finally never to carry a mercurial course beyond a reasonable time, say five or six weeks, or in some cases of secondary symptoms two months. If under all these precautions the disease, be it a primary sore or an eruption, get no better or become worse, let the surgeon wash his hands of mercury altogether, and try other and less formidable weapons. The patient's general health should on no account be allowed to be *impaired* by mercury; if it is so he has had too much of that mineral, or too little assistance in the way of good diet and tonics. We shall return to this subject at another time.

### IX. Severe Case of Hydrocephalus terminating in Recovery.*

In a paper by Dr. Graves, noticed in the present Periscope, there is a very philosophical analysis of a case of hydrocephalus by that able physician. The case before us may usefully be added to that, in order to elucidate some points connected with the disease.

Practitioners are too apt to regard hydrocephalus as a disease, the symptoms of which are tolerably distinct and decisive, and not as a term applied to an affection varying greatly in its different stages, both in point of nature and of character. It has long been a contested point, whether serum once thrown out into the ventricles, is or is not absorbed. Leaving this to be settled by those who feel competent to settle it, we may remark, that much of the discrepancy of opinion has arisen from the opposite parties not having limited their facts to cases of the same disease, in the same stage. When we consider, on the one hand, that children have died with all the symptoms of pressure on the brain, and, after death, no cause of pressure has been discovered;—and on the other, that children presenting such symptoms have been relieved from them by tonic or even stimulating treatment, we must feel how difficult it is to decide on the existence of effusion. We are driven under such circumstances to the rigid observation of facts.

*Case.* W. D. C. æt. 20 mens. became the patient of the late Mr. Reay, of Liverpool, on the 24th of April, 1830, labouring under a slight remittent febrile attack, with some cough and occasional fits of screaming. Mercurial purgatives were given, but squinting supervened, and on May 14, Dr. Traill was called in. The child was now very hot, with a rapid pulse; the alvine discharges ill digested and extremely offensive; the abdomen, though not tumid, felt *doughy* or inelastic; the tongue was furred; there was no marked impatience of light, the pupils regularly contracted, but the child occasionally screamed, without apparent cause, and the urine was scanty. He had cut all the incisors, the canine teeth, and four of the first molares; smart doses of calomel and jalap, with a mixture containing squill, were prescribed, while the head was ordered to be kept cool by an evaporating lotion. At 1 a. m., of the 16th the child had a severe convulsive fit. *Gums divided*

* Provincial Trans. Vol. I.

*over the molares—enemata—leeches to the temples—warm-bath—castor-oil.* On the 17th, more symptoms of cerebral affection—impatience of light; frequent screaming; convulsive twitches of the limbs—*leeches, blister between the shoulders, evaporating lotion to the head, calomel and jalap in repeated doses.* The blister was dressed with the ung. hyd. On the 19th there was strong strabismus; pupils much dilated, and nearly insensible to light. Yesterday and to-day all the other bad symptoms were increased; screaming more frequent; left side seemed paralytic, while the limbs on the right side were frequently and convulsively agitated. *Hyd. c̄ cret. thrice daily.* On the 21st the blister was repeated, and castor-oil given. On the 22d, the pulse which had previously been generally rapid, was now between 70 and 80. *Cold applications to the head omitted.* On the 23d the urine was nearly suppressed, the eyes insensible to light. *Calomel and jalap—nitre-whey.* On the 24th, moaning and screaming, urine very scanty, one side (not stated which) quite paralytic, the other constantly affected with convulsive twitches. *Blister with ung. hyd. repeated—castor-oil, and enemata.* On the 25th, the child began to be under the influence of mercury, and the blistered surface was highly inflamed; convulsive motions less violent. From this time he continued slowly to improve. On the 1st of June, strabismus still continuing, the eyes appearing to be yet insensible to light, and the pulse being below 70, rather irregular, diuretics were continued, and a small blister was applied to the vertex, over the fontanelle. On the 4th, the urinary secretion was copious and the strabismus diminished. On the 5th the blister was repeated. Soon after the 11th July he was in vigorous health, and he remains free from complaint.

It is interesting to trace the successive phases of this affection—first, derangement of the bowels, pyrexia, slight affection of the thoracic organs, marked by cough: and of the head, evidenced by screaming—secondly, the head more decidedly affected, marked by increase of fever, a tendency to squinting, occasional screaming; probably inflammatory action of the arachnoid or substance of the brain was now going on—thirdly, increase of cephalic affection, shewn by convulsions and convulsive twitchings of the limbs, impatience of light, more frequent screaming; probably effusion was now commencing, and the vessels were much loaded, for convulsions, after injuries of the head, are generally found to depend on moderate pressure—fourthly, symptoms of decided pressure, evinced by the subsidence of the pyrexia and the paralysis. He who carefully considers cases in this manner—who groups the symptoms, and calculates, not merely what the name of the disease is, but what are the particular functional conditions or organic changes producing those groups, will be the philosophical and successful practitioner.

An obvious and an interesting fact in the preceding case, is the subsidence of the symptoms on the appearance of mercurialization. We subjoin the following observations of Dr. Traill.

"In the treatment of such cases, I have, for several years, discontinued the application of severe blistering to the scalp, which was once a very general practice; from having observed little benefit from that mode of treatment, and having, in some cases, thought that it tended to aggravate the symptoms. I have, of late, applied the blisters more frequently to the nape of the neck, under the impression that the inflammatory state of the brain was more certainly combated by deriving the fluids *from the head,* than by increasing the activity of the vessels of the scalp; while the application of cooling lotions, at the nearest possible point to the seat of the inflammation, has appeared to me a more successful method of treating this very fatal disease. With this mode of local treatment, I have long been in the habit of conjoining the abstraction of blood, either by leeches or the lancet, according to the age and strength of the patient; and, as the influence of mercurials in controlling inflammation, and in promoting absorption, appears to me well established, I usually endeavour to induce a constitutional effect, in such cases, as speedily as possible, both by giving it internally, and applying it as a dressing to the vesicated surfaces. Indeed, I believe that mercury will, in this disease in particular, enter the system much more readily by cutaneous absorption than by the lacteals. As pressure on the brain would seem more quickly to paralyze activity of the absorbents of the alimentary canal than of the *dermoid* surface, probably because of the immediate dependence of the former on the great sympathetic nerve. In the case about to be given, these were the indications which were chiefly followed."

We would merely add this remark. Delightful as a cure must be, it is precarious; and we stand a better chance of preventing effusion than of curing it. As soon, then as there are symptoms of vascular action in the brain, displayed in a tendency to strabismus, screaming, pain in the head, and fever, it is better to push the mercury, conjoined with purgation, and depletion or counter-irritation, as circumstances require. This has been, with us, more successful than waiting a little longer before the mercurial practice is adopted.

---

### X. Case of Umbilical Hernia, in which the Liver formed Part of the Sac.

This case is related by Dr. M'Lean, of Kilmalcolm, in our Glasgow contemporary for July.

In 1820, his opinion was requested regarding a tumor, situated immediately over the umbilical region of a child, aged nine months, which was first observed a short time after birth, and which had gradually increased in size. On examination, he found the umbilicus filled with a hard body, which yielded partially to pressure, and became somewhat diminished in size, apparently from the yielding of its contents, although the great bulk of the tumour still remained. Its external covering was of an ash-grey colour, and for eight or ten days before the child died, it became partially ulcerated and discharged a thin fœtid sanies. The child always cried bitterly when the swelling was handled, pressure apparently producing considerable pain. The bowels were very much constipated, the dejections dark and offensive, and occasionally of a clayey colour and consistence. The appetite was impaired, and there was sometimes vomiting after taking any solid food, although no bilious or feculent discharge ever took place from the stomach. The child was ordered frequent doses of calomel and rhubarb, with other mild laxatives, to keep the bowels as regular as possible, but this was very difficult, there being much tendency to costiveness present. Warm fomentations were frequently applied to the tumour. By degrees it became softer, and fluctuation was distinct; but it was not considered advisable to open it, as the child was evidently fast sinking. Pressure on the tumour at this time had the effect of bringing on convulsions, and four days after fluctuation was perceived, the infant died in a convulsive paroxysm.

*Dissection.* The tumour was an umbilical hernia, and the sac contained a large portion of the liver, indurated and covered with numerous tubera, and about three inches of omentum, along with a portion of ileon. These parts were all completely matted together, and at the lower part of the sac, surrounding the lobulus spigelii, there was a considerable quantity of purulent matter. Nothing like stricture was observed, but from the strong adhesions which existed between all the parts contained in the hernial sac, considerable obstruction to the action of the intestines would no doubt be the consequence. The stomach and intestines exhibited traces of inflammation apparently of long continuance, and some portions of their peritoneal surface shewed numerous small dark-coloured spots. The other viscera were healthy.

Gay, Nourse, and Bohnius, are mentioned by Pott to have met with cases, in which the liver was contained in an umbilical hernia.

---

### XI. AFFECTIONS OF THE SEPTUM OF THE NOSE.*

Mr. Fleming, surgeon to St. Anne's Parochial Dispensary, has communicated a paper on some affections of the nasal septum to our valuable contemporary of Dublin. The particular affections which he describes are two—bloody tumour of the septum, and abscess. We suppose that the pugnacious propensities of our Hibernian countrymen would render the former accident frequent, and surgery is probably indebted to the shillelah, for some of Mr. Fleming's elucidations.

*Bloody Tumour of the Septum.*

This is always, as Mr. F. believes, the result of injury. These tumours resemble common ecchymosis in other parts of the body, and are often as quickly formed, generally within the first few hours from the occurrence of the accident: they usually occupy both sides of the septum, but may be confined to one; their extent and form are very variable, the mucous membrane in some cases presenting only a flattened elevation, appearing as if raised by an uniform effusion underneath, and in others being distended to a greater or less degree. There is considerable resistance in their feel, and this, combined with extreme tension, and surrounding hardness, renders it proportionably difficult to ascertain the existence of a fluid within. Mr. F. has always been able to see them by gently pressing the tip of the nose, and dilating the nares; their colour is of a dark purple, and they present a smooth and glossy appearance: their connexion with the septum is by a broad base, with abrupt boundaries. The principal symptoms complained of by the patient, are a general fulness and stuffing of the nares, proportioned to the extent of the effusion.

Mr. F. relates a case in which he was obliged to puncture the swelling. The patient, a gentleman, was struck on the nose by his horse's head while hunting. The nose bled much at the time, but Mr. F. was not called to him till the following day, when he complained greatly of obstruction within the nostril. On throwing back the head, and gently pressing the tip of the nose, each opening of the nostrils presented a tumour, tense, shining, and of a dark purple colour, nearly filling its calibre; each tumour could be distinctly traced along its outer side, with a probe, passing insensibly, by a broad base, upwards and backwards towards the septum; this appeared to form a partition between them, although a communication was suspected from the effects produced by the alternate pressure of the finger passed into either nostril, for by this means, the tumour on the opposite side was rendered fuller and more prominent. By the same manipulation, the existence of a fluid within was clearly ascertained, particularly if the nose, at the same time, was grasped at its upper part, by the fingers of the opposite hand. In consequence of the extreme local suffering, Mr. F. punctured the tumour in the right nostril with a lancet. The effect was instantaneous relief. A quantity of blood, half fluid and half coagulated, escaped, and by pressure, both tumours were evacuated through the same opening, and subsided considerably: a good deal of diffused hardness and tumefaction yet remained, which prevented any accurate examination of the septum. Cooling applications, leeches, rest, and the use of some gentle saline aperients, were directed. For many days a fulness and tenderness on each side of the septum existed, in other respects no remarkable occurrence took place in the progress of the case towards its ultimate cure.

Mr. F. observes that, in the treatment of these cases, we must be guided by ordinary principles, and that the necessity for an incision must be very rare.

*Abscess of the Septum.*

"Abscesses of the septum are then occasionally met with as the result of injury. As such they may be acute or chronic. They may also arise independent of that cause, in which case

* Dublin Journ. No. X.

they appear frequently to be connected with some scrofulous disposition in the constitution, or with the presence of some of the exanthemata, as variola, measles, scarlatina. The nature of the injury to the nose likely to produce abscesses of the septum varies. I think, however, they occur often where there is an accompanying wound of the integuments, and where that wound is situated near the lower extremities of the nasal bones, with or without injury to them. It usually happens, that the abscess is fully formed, when the surgeon is applied to, or (if he have had an opportunity of watching the case from the commencement) that the exact situation of the inflammation escapes his observation, until it has advanced too far to prevent suppuration. In those abscesses, the integuments of the nose generally partake of the inflammation. Though not always discoloured, they are œdematous, and tender on pressure. The pituitary membrane is inflamed throughout, and that portion of it covering the septum is particularly turgid. Its natural secretion is also suppressed, and should any external wound be present, it looks angry and irritable. The constitution generally sympathises, and ordinary feverish excitement prevails. At an earlier or later period, matter is formed under the mucous membrane, occupying either or both sides, usually both; and in proportion to the extent of the effusion, there is a tumour, more or less prominent, in either or both nostrils, producing corresponding obstruction. The pain, as we might have anticipated, spreads along the mucous membrane to the frontal sinuses, and lacrymal passages; hence the lacrymation and uneasy sensations in these parts complained of by the patients. It likewise occasionally spreads downwards; hence tumefaction of the upper lip and lower margin of the septum. The appearance of these tumours is remarkable. They are smooth and shining, and of a bright red colour; very tender on pressure, and give a distinct sense of fluctuation. They are somewhat fixed, and do not appear influenced by the ordinary acts of respiration. Their connexion with the septum is by an extensive base, and in every case I have seen, there has been a communication between those on opposite sides."

*Case.* A coachman, æt 40, fell and struck his nose against the edge of the curb-stone. A wound was produced. It was dressed, and, for eight or ten days nothing unusual occurred. The wound then became extremely painful, the pain extended to the neighbouring parts, the nostrils grew obstructed, and there was febrile disturbance. Mr. F. now saw the patient. The nose was enlarged from subcutaneous effusion, which implicated, with cutaneous redness, the eyelids, lower part of the forehead, and upper lip. There was much lacrymation, much pain on pressure, ulceration of the wound, exposing the nasal bones denuded, and a probe could be passed on either side of the septum for some distance upwards, backwards, or downwards. The nostrils were blocked up by two highly-vascular tumours, which projected considerably beyond their margin. These tumours were tense and polished on their surface, and so fully occupied the nostril, that they were almost fixed and unaffected by the ordinary act of respiration. By firmly compressing the nose at its lower part, a thin, sero-purulent fluid could be expressed through the ulcer; by having recourse to the same means of compression at the upper part, the tumours below were rendered more tense and projecting, and by alternate movement, no doubt could be entertained as to the existence of a fluid within them. The outer boundary of each was defined, and could be traced with a probe towards the median line, where the septum separated them. Mr. F. made an opening with a lancet into the tumour, in the right nostril, when a large quantity of a thin purulent fluid escaped, and both the tumours subsided, leaving the mucous membrane in loose sacculi, on each side of the septum. A dossil of lint was introduced into the opening, and ordinary local and general remedies directed. Great relief was experienced from the operation. Much difficulty was experienced in keeping the opening free, and six weeks elapsed before the wound was healed. Twelve months after the accident, Mr. Fleming met the patient, and examined the nose. No exfoliation had taken place, but occasional uneasiness was felt in the cicatrix of the original wound. The central portion of the cartilaginous septum appeared to have been absorbed, and to have admitted of the adhesion of the opposite surfaces of the mucous membrane to each other. This had produced a change in the form of the nose, the dorsum having fallen in, in a slight degree, between the tip and the extremities of the nasal bones. No other peculiarities were to be observed.

"Abscesses of the septum are always to be looked on by the surgeon with anxiety. He ought to have recourse to every means in his power from the date of the injury to the nose, to prevent their formation, and when the slightest grounds exist for suspecting the presence of matter, he should not lose time in making an opening to evacuate it. This is the only chance the patient has of escaping a tedious disease, and ultimate deformity, from the bones or cartilage partaking of it. The thickened state of the mucous membrane is to be borne in recollection in puncturing those tumours, and in their future treatment. They should be rendered as tense as possible by firmly grasping the upper part of the nose, and in the subsequent visits the opening should be freed, as the fulness of the tumours may indicate the fresh accumulation of fluid. The discharge is generally of a thin, sero-purulent nature, and in the progress of the case I have remarked, that it assumes a glairy consistence. The mucous membrane is slow in recovering its healthy condition. It is, however, materially assisted by different lotions: in the inflammatory stage, those containing lead and zinc are grateful; in the chronic, the black and yellow mercurial washes, and the diluted citrine and zinc ointments, will be found beneficial. The general, local, or constitutional treatment does not require any particular comment."

Mr. Fleming appears to consider as a peculiarity, the discharge assuming a glairy character in the progress of the case. This is observed in all abscesses. As the cavity contracts, and the cure is effected, the discharge invariably passes from the condition of purulent to glairy, and from that to serous.

Mr. Fleming has also seen instances of abscess arising spontaneously. They are seldom suspected by patient or practitioner, till fully formed. He has never seen the outer parietes of the nose engaged in these abscesses. With the following quotation we will drop the subject.

"Their appearance is natural, and unless deformity exist from the extent of the abscess, we are obliged to examine the nares for their detection. Here the only peculiarities they possess different from the symptomatic or acute, are, that there is a less shade of redness in their colour, that they are less tense, and that they bear more pressure without pain. I think also, they are much more extensive, and more likely to occur singly on either side of the septum. I have met with the case of a countryman, where not only each cavity of the nostril was occupied by a tumour, but there was considerable protrusion of the upper lip, and on everting it, an abscess exactly resembling in appearance and situation a common gum-boil, was found at the root of the septum, which, on being opened, gave exit to a large quantity of thin, purulent fluid, and caused the subsidence of all the swellings. Again, I have had under my care a young lady, with an abscess about the size of a spanish nut, occupying only one side of the septum, about an inch or an inch and a half from its anterior margin. The history of the first of these cases was most confused and unsatisfactory. The obstruction in the nose had been felt for an indefinite period beforehand, and with so little uneasiness or pain was it accompanied, that I really believe, were it not for the deformity, no application would have been made for relief. It had been considered in the neigbourhood of the character of polypus. Its termination I am not aware of, I am only satisfied of its nature.

The account which the lady, who was the subject of the second case, gave of herself, was as follows: when travelling in England about a month before, without any previous uneasiness in the nose, she suddenly perceived a most disagreeble noisome smell, which, at the moment, she was inclined to attribute to some accidental cause in her apartment, at the hotel at which she stopped. She could not, however, get rid of the sensation, and although it varied in its pungency, it was more or less constant. Under those circumstances, she applied to me. In the examination of the nares, I could only observe the tumour I mention in connexion with the septum. It had a fistulous opening, through which oozed out a thin fluid, having the fetid odour complained of. Some time elapsed before it subsided. It ultimately, however, did subside, and was most benefited by the occasional injection of a strong solution of the nitrate of silver, and the administration of mild alteratives."

---

XII. Dr. Ayre on Cholera. 1 Vol. 8vo, pp. 167, Sept. 1833.

Though we received this work at the eleventh hour, we are unwilling to let it pass unnoticed in the present number. The epidemic has paid us another smart visitation, and how long it may continue to recur is yet a matter of conjecture. Dr. Ayre's attention has long been directed to cholera, both the common annual disease and the malignant, or epidemic form of the malady. His opinions and practice are, therefore, entitled to the serious consideration of the profession.

It is curious that, in the year 1817, (the date of the great outbreak of the disease in India,) cholera was epidemic in England, and was described, in 1818, by Dr. Ayre himself. Speaking of severe cases and types, he observes—

"This was especially the case in the year 1817, when it prevailed as an epidemic; and when, amongst several of a milder grade, there were a great many cases of it, in which, as a part of the complaint, there arose a condition of the system which had rarely before been witnessed, and never, perhaps, with such distinctness. This condition consisted in a great oppression or collapse of the vital powers, and disturbance in the nervous and sanguiferous systems; and characterised by a deathlike coldness and lividness of the surface, by a feeble pulse, a sunken countenance, violent vomiting, with a manifest diminution or suspension in the secretion of the bile. When these symptoms subsided spontaneously, and the patient survived them, a state of fever supervened, which, after running a course of one or two weeks, frequently assumed a typhoid form."

Dr. Ayre considers the Indian and the English disease to be identical—the epidemic character rendering the latter more fatal and formidable than when in the sporadic form. In a chapter on the remote causes, he very ably advocates the epidemic and non-contagious nature of the disease; but enough has been said on that subject, and we hear little or nothing now about contagion. In some of our hospitals, when a case occurs in a ward, the patient is not even removed to a separate ward, but treated till death or recovery takes place, like any other patient, and without exciting the least alarm of infection among the residents of the same ward! Who would have believed this in the beginning of last year!!

One of Dr. Ayre's chapters is on the nature or pathology of cholera. Dr. Ayre thinks that the collapse cannot be accounted for, by any supposed change in the composition of the blood, or any loss of certain constituent parts of that important fluid. On the latter point we certainly disagree with our author. The more we see of cholera, the more we are convinced that the disease, in all its worst forms, is a *serous hæmorrhage* from the mucous membrane of the alimentary canal. We quite agree with Dr. Ayre, that—"The premonitory diarrhœa, which, if neglected, runs into the disease, and in a multitude of cases forms the incipient stage of it, is often directly induced by irregularities of diet, as a large indigestible meal, and may be arrested, and the full development of the true disease prevented, by remedies of a common kind, and which have confessedly no power to prevent, or correct any *change* in the condition of the blood." Granted. We are quite convinced that, be the primary cause what it may—malaria, atmospheric constitution, or contagion if you please—the effect is a diarrhœa, which diarrhœa is the first stage of the serous hæmorrhage—and collapse is the natural consequence of the loss of the serous part of the blood. This loss is, we verily believe, worse than an equal loss of pure blood from the bowels; because, in common hæmorrhage, what blood is left, is fluid and good; whereas, in cholera it is thick and unfit to circulate or support life. The pith of Dr. Ayre's theory is found in the first of his inferences.

"1st. That the cholera morbus essentially consists in an interruption, and, in its malignant form, in a sudden and entire cessation of the secretion of the liver, and primarily, as the result of it, of a congestion of the portal circle, or secretory system of veins of the liver; and, in the malignant kind, successively of those veins of the abdominal viscera and vertebral column, whose venous circulation is associated with them."

We need hardly remark that this is almost precisely the theory broached nearly 20 years ago, by the editor of this Journal, as any one may ascertain by referring to the chapter on cholera morbus, or mort de chien, in first and all subsequent editions of "the Influence of Tropical Climates, &c." We shall quote the ninth inference respecting the remote cause.

"9th. That the remote cause of the foregoing pathological conditions consists in a morbid irritation primarily set up in the stomach and bowels by a certain malaria, assisted by unwholesome ingesta;—that the malaria is of a specific nature, and generated in certain localities conspicuous for defective drainage and other definite peculiarities, and modified or wrought into its state of malignancy by certain concurrent, but unappreciable conditions of the atmosphere;—that the specific malaria thus modified exerts its influence chiefly in the localities where it is generated, and where, from its concentration, it is embued with the most power; and, lastly, that it affects within the range of its influence almost exclusively those only of the community in whom a predisposition is induced by the habitual disuse of animal food, and by the derangement of the stomach and of the system, and which has resulted from an exclusive, and, therefore, inordinate use of a vegetable and ascescent diet."

The above is also the etiology maintained by Dr. Johnson twenty years ago, in the volume alluded to.

TREATMENT.

In the premonitory diarrhœa, or first stage of the disease, Dr. Ayre gives one grain of calomel united with two or three drops of laudanum, repeated every hour, or every half hour, for six or eight successive times—and then, every six hours, or twice a day, for a short period, directing rice to be substituted for bread or potatoes, and to take some animal food. The patients were directed, if the disease assumed a more serious form, to begin immediately with the calomel and laudanum, every five or ten minutes, and to acquaint him with the change. Very few treated in this manner lapsed into the worst stages of the disease.

In the stage of *collapse*, Dr. Ayre usually gave a grain of calomel, with one drop of Battley's sedative liquor, in a tea-spoonful of cold water, every five or ten minutes, according to the urgency of the case.

"In a few cases of extreme severity, I gave two grains of calomel every five minutes for an hour or two, and then resumed the ordinary dose of one grain. In giving this medicine, no other limit is required to be set to its use than that which the state of congestion or collapse imposes; for pending its duration the medicine must be uninterruptedly continued, watching, at the same time, the decline of the disease, and widening the intervals of giving the medicine to ten, fifteen, and twenty minutes, until it becomes evident, by the symptoms, that this stage of the disease has passed away; for the mercurial effect of ptyalism, which is of no advantage to the complaint, will be excited if the medicine be used to any extent, either before the collapse has commenced, or after it is removed. In a very few cases only were there any ptyalism produced, and in them it was inconsiderable, and chiefly confined to the slighter kinds, and to those which were treated as premonitory, and not reported."

In cases of the most intense severity, and where the collapse had been greatly protracted, with profuse discharges from the bowels, much advantage was derived from starch and broth glysters, with opium. Except cataplasms of mustard to the abdomen, with bags of hot sand to the feet, few other auxiliaries were used. Indeed we have every reason to believe that thousands of lives have been lost from officious interference, and a farrago of heterogeneous and stimulating remedies, in this formidable complaint.

We need not dwell on the consecutive fever. It is always symptomatic of inflammation, and generally of the stomach or bowels. Leeches to the epigastrium, and antiphlogistic treatment are the best practice.

In conclusion, we believe this plan of Dr. Ayre's to be, perhaps, the best as it is the simplest of any now in practice. With some modification, it is that which we have employed in the epidemic of this year.

---

### XIII. Mr. Carmichael on Inflammatory Affections of the Brain and its Membranes.*

This paper consists essentially of cases, chiefly detailed from memory, and the apothecary's file of prescriptions. This is not an unexceptionable mode of recording facts. Appended to the paper are "a few desultory observations," nearly as long as the paper itself.

The object of M. Carmichael is stated to be—"to give a few illustrations of the variety of symptoms, which an inflammatory state of the brain, or its investing membranes, is capable of producing." The first illustration is the following.

*Case* 1. "On the 18th of March last, I was called at a very early hour in the morning, to see Mr. H——, a robust, full man, about 38 years of age; I found him walking about his drawing-room, apparently in great anguish. He complained of violent pain in his ear, shooting upwards along the side of his head, which had affected him more or less during the preceding fortnight. He stated that he had been under the care of Mr. Barker, of Great Britain Street, but that he had attended little to his advice, as he not only went to his office almost every day, but dined and supped out, and even had gone a hunting." The pulse was quick, the tongue white, the countenance indicative of great pain and distress.

Mr. C. considered the case one of inflammation, ordered 16 leeches near the ear, cathartic medicine containing tartarized antimony to be taken every hour. In the evening the patient was better, and Mr. C. did not see him again till the 20th.

He was then suffering from pain, though less than before. The expression of the countenance was bad, and he complained much of sickness of stomach. Ordered—*Cal.* gr. iij.—*pulv. ipec. comp.* gr. x., *followed by cathartic medicine. A blister behind the ear.*

On the 21st the pain continued—he had had a bad night—pulse frequent and feeble.—*Cal.* gr. j. *omni horâ.*

22d. Better—pulse only 80—stomach a good deal disordered still. It was therefore determined to give him occasionally, in addition to the other means, a couple of spoonsful of a cold infusion of bark, in a state of effervescence, and at night, in order to induce sleep, a bolus, composed of three grains of ext. of hyosciamus, with two grains of camphor, was ordered.

Mr. Carmichael's attendance was now discontinued, and another gentleman, Mr. Irvine, took charge of the patient. On the 24th, however, Mr. Irvine again called in Mr. Carmichael to see the patient who was comatose. He had been ordered on the preceding night the fourth of a grain of the acetate of morphia. A mustard emetic was given, under an erroneous suspicion of a mistake hav-

---

* Dublin Journ. No. X.

ing been made in the medicine—16oz. of blood were abstracted from the temporal artery—a blister was applied to the head, a sinapism to the epigastrium, and a cathartic enema administered, but in spite of all these means the patient was seized with strong convulsions, and died comatose, and with stertor, during the night.

*Dissection.*—"An extensive purulent secretion, of a pale greenish colour, was found between the arachnoid membrane and pia mater, where these membranes invest the lower surface of the middle lobe of the right hemisphere, and the lower and lateral surface of the middle lobe of the left side, extending over a portion of the anterior lobe of the same side. On slicing the brain, the substance appeared studded with many red points; on opening the lateral ventricles, about half an ounce of fluid was found; choroid plexus, not more vascular than usual; some purulent secretion was found on superior surface of cerebellum of left side, also on the pons varolii and commencement of medulla oblongata.

The pia mater between the convolutions of cerebrum on left side, was as red as the conjunctiva of the eye in inflammation of that membrane.

The petrous portions of temporal bones were perfectly sound; nor did the neurilema of the seventh pair of nerves appear inflamed, notwithstanding the violent pain complained of in the right ear, and the last day in the left."

We must confess that, great as is our respect for the talents and attainments of the gentlemen concerned, we do not approve of the treatment pursued in the preceding case. A plethoric person has violent pain in the side of the head, which has lasted for some time and been aggravated by imprudences, and is attended with loaded tongue and quick pulse. He is relieved by leeches and cathartics. We would rather have bled the patient than leeched him; but let that pass. Two days afterwards he is again found suffering from pain, and he has sickness of stomach. Under these circumstances, he is ordered three grains of calomel and ten of Dover's powder, with cathartics and a blister. Why was opium given? Under such circumstances bleeding, or cupping would surely have been preferable to the blister, and an active system of purgation with calomel would, in our humble opinion, have been the most appropriate remedies. He is now, however, put on mercury and improves a little, when he is ordered bark, with hyoscinamus and camphor. Again after this narcotics are given. He dies with purulent effusion between the membranes. We repeat that, we think the patient would have had a better chance of recovery, if he had been more actively treated in the first instance, and more antiphlogistically throughout. We have noticed the case with the view of cautioning practitioners, for, unfortunately we have seen others not very dissimilar.

The next case is intended to illustrate the powers and the use of a tartar emetic plaster.

*Case* 2. Early in May last, Mr. C. was called to see a gentleman, aged 70, on account of intense head-ache, which he had suffered from for some time, and an anthrax on his neck. The tongue was foul, and the digestive organs much deranged; he was subject to vertigo and tottering in the limbs.

The anthrax was scarified, the bowels opened daily with blue pill and ext. of colocynth, and small doses of quinine with sulphuric acid prescribed. The anthrax did well, but the head-aches continued severe, the attacks of vertigo were frequent, and the giddiness of the limbs increased. Leeches were ordered to the temples, cold applications to the head, and a blister to the nape of the neck, followed by the use of tartar-emetic ointment; while blue-pill, combined with colocynth, was given with the double view of acting on the bowels, and of exciting such a mercurial action as would tend to check any inflammation that might exist in the brain or its membranes. The headaches under this system were soon lessened, but he began to loose his speech; his memory failed him so far that he forgot the most common words, and from being intelligent and active, became quite imbecile in intellect and unable to move. He now continued in a lethargic state in bed. Tempted by the result of another case Mr. Carmichael determined to apply a plaster of tartar-emetic to the occipital region. In 24 hours considerable pain was excited, but the plaster was kept on for 64 hours, when it was found to have produced an extensive sloughy-looking sore. The patient's recollection and speech now returned, almost suddenly, and after this time he gradually recovered, and continues well.

The case is practically interesting, and should be kept in mind. We think that Mr. Carmichael should rather have avoided the quina in the first instance. Anthrax or boils occurring with headaches seldom require tonics, indeed the latter are almost always injurious in such circumstances. The most that should be done is to give nutriment without stimulus.

The third case presents nothing of interest. The fourth has all the characters of mania following injury, and probably produced by chronic inflammation of the brain or its meninges, the consequence of the injury. It is interesting and we will give some account of it.

*Case.* A gentleman, about 70 years of age, of rather a full habit, and subject to gout, was thrown from a gig, and received a severe contusion on the head. He slowly recovered from the fall, and his manner was observed to be greatly altered. In spite of the advice of his medical attendant, he lived freely. In the last days of May, he was attacked with sudden delirium, increased vascular action, and nervous excitement, great inquietude, constantly kicking the bed-clothes off by means of the feet, tearing off his shirt, and getting out of bed and in again; suppression of the biliary, intestinal, and urinary secretions; a high degree of cerebral excitement, evinced by hot scalp and unnatural fulness of feature, as well as by the lively eye. On the third day of the attack, Mr. Carmichael saw him. The symptoms were such as have been described—the pulse 120, with some degree of sharpness — the bowels not moved by large doses of calomel and croton oil.

Mr. C. concluded that there had been chronic inflammation of the brain and its membranes, and that a more acute attack had supervened. He proposed venesection, to be followed by mercurialization as rapidly as possible. Dr. Wright observed that the patient had been bled moderately at the commencement of the attack, but that immediate prostration had succeeded. However, 12 ozs. of blood were abstracted from the arm, and eight grains of calomel given. In the course of three hours, the medicine had a powerful effect on his bowels, although previous to the bleeding it had resisted treble the quantity. After his bowels had been affected he became cold and feeble, with a small wiry pulse. In consequence an anodyne draught was given to him, which restored his pulse, and threw him into a sound sleep for several hours, a refreshment he had not enjoyed for many nights, and he awoke in the morning able to answer questions with distinctness. The further treatment pursued was calomel, so as to affect the system—blistering of the occiput, and cold applications to the forehead and upper part of the head. On the 3d June, 10 ozs. of blood were again abstracted. On the 9th the mercury was discontinued. At times, there was sinking, with irregularity of the pulse, which was always restored by small doses of morphia. On the 12th active measures were discontinued, and equal parts of camphor mixture and infusion of valerian were given three or four times daily.

The progress of the case may be explained, by the following extract of a letter from Dr. Wright to Mr. Carmichael.

"On the fourth day, the patient got all the unfavourable symptoms of the last stage of typhus fever—the pulse sunk, he became comatose, with subsultus tendinum, involuntary evacuation, catching at imaginary objects, picking the bed-clothes, hiccup, tremulous motion of the muscles, falling of the jaw, with stertor, and hemorrhage from the bowels.' [This symptom was owing to the mercury, which was then discontinued.] 'On the seventh day there was a crisis; the symptoms generally suffered a remission, but on the eighth day there was an increase of the symptoms again, or rather a return of the former symptoms in a modified degree; there now came on a convulsive starting, and involuntary throwing about of the hands. On the fourteenth day another favourable change took place; there was some little looking up during the week following, and on the 21st day there was another marked crisis, and from that day his health has been gradually improving. He is daily gaining strength, but I am sorry to say the mind does not improve; he labours under marked imbecility. Although the lawn of —, and his own improvements, are before him, which he notices and talks of, he thinks he is at Newtownbarry: he is quite cheerful and merry, but will not dress himself properly, and he must be humoured, or he would become restive; sometimes he converses rationally for a few minutes, but soon flies off to some fancied subject."

We would recommend Mr. Carmichael to be more methodical and more concise in the narration of his cases. They are sadly diffuse. Indeed, if we could venture to hint a fault in the papers of our excellent contemporary, it would be such an one as we have noticed in Mr. Carmichael. The warm imagination and the generous fancy of Irishmen are seldom trammelled by the cold and sullen rules of exact composition.

As an instance of the picturesque style of their facts, we may adduce the opening of Mr. Carmichael's fifth case. The cumulation of epithets, and the liveliness of the description, cannot fail to be perceptible to those most unaccustomed to the analysis of literary efforts.

"On the 6th of May last, a drunken fellow of the name of Michael Reilly, was admitted into the Richmond Hospital, under Dr. Hutton's care, with his ear literally ground off by the friction of the wheel of a dray, which he was attempting to drive while in a state of most deplorable intoxication.

Not only was the entire of the left ear destroyed, but a frightful lacerated wound, at least five inches in diameter, on the side of the head, exposed large portions of the temporal and parietal bones. The squamous suture was as clearly seen as in a well-prepared cranium for lecture, and the circumvolutions of the wheel had left a distinct and deep track upon the parietal. Uppon being asked how it was possible that he could have been reduced to such a state of helplessness as to lie quietly on his dray while he felt the wheel grinding off his ear, he very coolly replied, while scratching the surviving ear; 'Why thin I'm thinking, your honour, that somehow or other I must have been purty well *tossicated* at the time; but I believe it was more the fault of this big coat o'mine, though faith, as you see Sir, *it* was sober enough any how. It caught the wheel or the wheel it, I wont say which; I was dragged on one side, my hat fell aff, and the wheel ground the ear aff o'me entirely!"

Much as we admire the ardour and the feeling of our brethren of the Green Isle, perhaps it would be well to sacrifice a little to sober perspicuity. They might reflect that, although an individual case, related in this graphic and easy manner, might excite a smile, and charm into perusal the unwilling scholar, yet that general medical literature of such a character would become most diffuse, most trifling, and most wearisome.

The remainder of the case which has illustrated these remarks, presents very little to detain the reader. Erysipelas followed the injury, and pain in the head, with a countenance expressive of internal distress, succeeded to the erysipelas, the patient was at times delirious, and complained of a sense of weight in the præcordia. These symptoms were relieved by mercury, given to the extent of affecting the mouth. The exposed bone subsequently exfoliated, but the wound healed, and the patient did well.

The sixth case is short, and may be given for the purpose of shewing the general character of the treatment recommended by Mr. Carmichael. It is such a case as probably occurs very frequently to all surgeons or physicians engaged in practice.

"Richard Hamill, aged 42, of an unhealthy aspect, was admitted 25th June last, into the Richmond Hospital, under Dr. Hutton's care. At the time of his admission he was unable to stand for any length of time, and tottered like a drunken man when he attempted to walk, so much so, that he had frequently fallen during the previous five weeks, the period of his illness. He had constant vertigo. His vision was very imperfect, objects appearing double, indistinct, and magnified. He complained also much of noises in his head. The sense of feeling was also injured, as well as the muscular powers, for he was incapable of grasping any object firmly. The treatment adopted was general blood-letting, purgatives, and counter-stimulants to the neck, and the shower-bath.

On the latter end of June, in going through the wards, I saw him for the first time, with Dr. Hutton, and we agreed to put him under the influence of mercury. One grain of calomel was ordered three times a day, and a tartar-emetic plaster to the back of his head. Under this plan, his amendment was decided and rapid, and he was discharged convalescent, on the 13th July, able to return to his work, that of a harness-maker, in the firm of an extensive coach-factory."

We imagine that there are few good practitioners in this country, who would not have adopted nearly similar treatment in a similar case. General bleeding, used with discretion—purgatives, especially of the mercurial kind, abstinence, rest, with cupping and counter-irritation, are the means very commonly and very effectually employed. If these means failed, mercury to affect the system would in all probability be resorted to, as it was by Mr. Carmichael.

"In the treatment of inflammatory affections of the brain and its membranes, next to blood-letting in efficacy stands, in my opinion, the mercurialization of the system. The beneficial effects of this process in stopping the progress of inflammation of membranous parts, is most satisfactorily demonstrated every day by the exhibition of mercury for iritis. Frequently in this affection, the pain, change of colour, and depositions of lymph on the iris, occasioned by the inflammation, begin to disappear even before the mercury has had time to evince its usual effects on the gums of the patient. Perhaps other agents may exert an equally specific (I use the word for want of a better) effect upon other membranes. Thus there are strong grounds for believing that terebinthinates exert a peculiar influence over inflammation of the mucous membrane, a familiar example of which we have in the effects of balsam copaivæ in gonorrhœal inflammation of the urethra; and I may also adduce the effects of turpentine in reducing the inflammation of the uterus and intestines, which attends, or rather constitutes, puerperal fever. But to return to mercury. This powerful instrument has not been made use of as it ought, for inflammation which succeeds to injuries of the head, and for which the trephine is so often vainly employed for the purpose of giving exit to matter, when the symptoms of a compressed brain follow those of inflammation, and which, in 99 cases out of 100, is found beyond the reach of operation, to be extensively diffused between the pia mater and arachnoid coat."

We have on several occasions directed particular attention to the employment of mercury, in the inflammation of the brain and its membranes that succeeds to injuries of the head. Our readers will find many practical remarks on this subject, and some instructive cases, in the reports from the Glasgow Infirmary, which have been reviewed in various numbers of this Journal. In the employment of mercury, then, in these circumstances, we cordially agree with Mr. Carmichael. The particular symptoms that call for its administration, and the extent to which it should be carried, must be left to the experience and the judgment of the surgeon.

But there is another point on which we do not agree with Mr. Carmichael. The following passage contains the opinions from which we dissent.

" In all surgical writers on the subject, we find futile attempts to establish an accurate diagnosis between the symptoms of concussion and compression. When a blow is inflicted on the head, more or less concussion of the brain must take place, whether or not the violence has been sufficiently great to rupture a blood-vessel on the moment. If a vessel should have been ruptured, giving rise to compression, the patient's symptoms are attributable to both causes. But concussion is always followed by more or less of inflammation or increased vascular action, and this inflammation, if not checked, may produce depositions, attended by coma, and all the symptoms of a compressed brain. The attempted diagnosis is therefore of no practical utility. The treatment is the same, except, perhaps, in the very rare instance, when the middle artery of the dura mater happens to be ruptured, in which case the immediate application of the trephine may be of use. It is needless to observe, that the preceding observations do not apply to cases of a depressed bone, where the cause of compression is obvious."

This doctrine appears to us to be equally dangerous and futile ; the logic is as inconsequential as the practice inculcated is pernicious. Mr. Carmichael argues, that because every violence sufficient to produce compression must also have produced concussion, and because concussion is liable to be followed by inflammation, and the inflammation to produce effusion and consequent compression, therefore the diagnosis is of no practical utility. How impotent this conclusion is, and how imperfect the premises, is shewn at once by this obvious reply. Although it may be true that compression from injury may not exist uncombined with concussion in the first instance, yet concussion may occur independently of compression; and although concussion may end in inflammation, and inflammation in compression, yet concussion does not always end in inflammation, and when it does so, the symptoms of compression produced by inflammation are very different from those which are independent of inflammation. Mr. Carmichael remarks that his reasoning does not apply to cases of depressed bone, because there " the cause of compression is obvious." To Mr. Carmichael depression of bone may always be obvious, but all men have not the opportunities of that gentleman, nor his powers of using them. We are not ashamed to confess that we have witnessed cases, where depression of bone was not obvious, although it existed and occasioned symptoms. We have little hesitation in offering our opinion that the broad symptoms of concussion and those of compression are essentially different, that they are not in general difficult of distinction by experienced surgeons, and that the diagnosis is of practical utility. It is true that many cases are of mixed character, but the very commixture of symptoms is itself a means of diagnosis.

The following remarks on head-ache are not undeserving of attention.

" There is no more difficult point in practice than to ascertain, at times, when a headach is symptomatic or idiopathic, particularly as it is allowed that head-achs, which at first occurred from sympathy with the stomach or other parts, may, from long continuance and severity, produce inflammation and its consequences in the brain or its membranes, and thus become idiopathic. This is too extensive a subject to engage on, in a paper which has already exceeded its just limits. Where doubts exist, a close attention to the juvantia et ledæntia of regimen and remedies, will greatly assist us in arriving at a correct judgment. This much I must remark ; that it appears to me that idiopathic head-achs, depending upon chronic meningitis, are more general than is usually imagined ; and that we often amend or cure the disease with remedies exhibited for the purpose of acting on the digestive organs. Thus mercury, either given as a purgative or an alterative, may remove chronic meningitis, the head-achs be consequently cured, and the practitioner confirmed in the truth of views which he had erroneously adopted.

Periodical returns of head-ache are usually esteemed nervous or sympathetic. But as it is the character of many painful affections of the nerves and their neurilema, to return at regular periods, the same regularity is likely to occur in similar affections of the brain and its membranes. These affections often depend upon inflammation, as is sufficiently illustrated in the fact that almost every one has experienced in his own person, of the periodical returns of tooth-ach, when the nerve is exposed by caries of a tooth, and thus with its neurilema consequently inflamed. The attacks of sciatica, a disease in which the neurilema of the largest nerve in the body has been often found on dissection thickened by deposition of lymph, and to exhibit other signs of inflammation, are remarkably periodical and regular in their returns, of which I, in my own person, have had most woeful experience."

As Mr. Carmichael observes, it is extremely difficult to decide, in some cases, whether head-ach is or is not dependent on affection of the encephalon or its membranes—whether it should or should not be treated by tonics. Besides the attention to the juvantia and ledæntia recommended by Mr. Carmichael, we would advise a strict inquiry into the state of all the functions. If there is any thing wrong, we cannot do ill in setting that right, and when the tongue is clean, the secretions all natural, the functions properly performed, we may begin to consider carefully our further line of practice. As a general rule, the headachs of the elderly and of the very young, are more frequently connected with inflammatory action within the cranium, than those of individuals between these extremes of age.

Mr. Carmichael speaks highly of the effects of the counter-irritation, by the ointment of tartar-emetic. All practical physicians and surgeons will agree with him. Blisters and the tartar-emetic, having a very different kind of action on the skin, appear to be adapted to different diseased conditions. Blisters occasion a large secretion of serum, and they generally answer best in inflammations of the serous and synovial membranes, after vascular depletion has checked, in a great measure, the vascular action. The tartar-emetic, on the other hand, agrees best in those chronic cases, where there is not such a tendency to serous secretion, and where, probably, the inflammatory action has a tendency to more destructive terminations. An interesting paper might be writen on the various modes of action of counter-irritants, the states to which they are applicable, and the diseased conditions they are severally best adapted to remedy.

We trust that no apology is necessary to Mr. Carmichael, for having criticised his opinions freely. He has himself exercised, and rightly exercised, a similar freedom towards others, and we feel assured that he appreciates the advantages of unrestrained discussion. For Mr. Carmichael's talents we entertain a high respect, and his work on the Venereal Disease is sufficient to stamp him as at once ingenious and bold. We hope to see more communications of his in our excellent contemporary.

---

### XIV. Principles and Practice of Obstetric Medicine. By David D. Davis, M.D. &c.

[Parts XXI. and XXII.]

### Nymphomania.

The fasciculi before us are principally occupied with the consideration of nymphomania. Disgusting and melancholy as the subject is, we believe that many erroneous notions on the nature and the treatment of the disease prevail, and, consequently, that a few pages devoted to its consideration are not altogether misemployed.

There cannot be a question that nymphomania is, in general, a symptom rather than a substantive disease. In the recent work of M. Dugés and Mad. Boivin, this view is embraced, and a very small space is allotted to the consideration of the malady. But, although this is generally true, it is probably not universally correct. A defective moral education, the influence of bad example or of prevalent corruption of manners, and particular circumstances affecting the passions of the individual, may give rise to the nymphomaniacal fury, independent of any thing which deserves to be regarded as local disease.

Unless we permit this view to be taken, we shall find it difficult to account for the greater prevalence of the affection, at one time and in one class, than at another time and in another class. The astonishing extent of prostitution, of every description, that was witnessed in the Roman Empire, under the imperial sway, may justly be considered as a species of nymphomania. Dr. Davis himself indulges in this supposition. He quotes the cases of Cleopatra, and of Messalina, "the beautiful empress of the weak and cruel Claudius." The "incomparable prostitutions" of the former, excited the amazement and fed the suspicions of Marc Antony, who, in a letter to Soranus, his physician, would seem to have hinted their dependence on disease. The lust of Messalina has been unhappily immortalized by Juvenal and Pliny. Verse and prose have consecrated the feats of this votary of Venus.

> "Tamen ultima cellam
> Clausit, adhuc ardens rigide tentigine vulvæ,
> Et resupina jacens multorum absorbuit ictus,
> Et lassata viris necdum satiata recessit."

Such is the damning, yet glowing description of the poet, whilst the philosopher bluntly remarks—"die ac nocte superavit quinto et vicessimo concubitu."

Had these instances been rare, they might safely have been considered cases of disease, and the philosophical historian would have gladly thrown the extenuation of bodily infirmity over the apparent vitiation of the mind. But they were not rare, and each succeeding emperor and empress would seem to have vied, in debauchery, with their ancestors and with each other. Faustina, the wife of the stoic Marcus Antoninus, an admirable Roman and a tender husband, was celebrated for her sensuality. *Conditiones* sibi et nauticas et gladiatorias elegit. A historian, Lampridius, explains the sort of merit that Faustina chose, and the conditions she exacted. What those conditions were, may perhaps be surmised, by reflecting on some of a similar character, which recommended those who could comply with them to Elagabalus. A dancer, a charioteer, and a barber, were promoted to offices of state, and introduced to the special favour of the sovereign, *enormitate membrorum.* We need not pursue the catalogue of queens, whose royalty was distinguished, in the period of the decline of the Empire, by the incredible excess of their venereal appetite. We repeat that a few and scattered instances might charitably and profitably be laid to complaints of the generative organs. But when every page of history is a record of lewdness, and appears little better than the chronicles of the brothel, we fear that the physician must abandon his claim to the case, and all must admit that the disease was of the mind, and that luxury, illimitable power, an enervating climate, and a foul religion, were productive of nymphomania on an imperial scale.

It must not be forgotten, that the female sex was not alone infected with this terrible abandonment of prudence and morality. The excesses of Faustina and Messalina were more than matched, by the beastly orgies of Commodus and Elagabalus. The decent remoteness from ordinary associations, enjoyed by the terms of a dead language, will scarcely serve to cover the astounding infamy of either of these princes. The latter was publicly married to men, and gloried in the title and the social position of the female—the former is faintly described in the Augustan history. Sororibus suis constupratis. Ipsas concubinas suas sub oculis suis stuprari jubebat. Nec irruentium in se juvenum carebat infamiâ, omni parte corporis atque ore in sexum utrumque pollutus. In either sex we observe, at this epoch, the same shameless depravity, the same disregard for instinct and reason, for the obvious intentions and commands of God, and the feelings and institutes of man. It was not, alas, nymphomania, but utter dissolution of morals.

We do not therefore agree with those who, like M. Dugès and Mad. Boivin, look on nymphomania as always a symptomatic, and never a substantive affection. We cannot doubt that a naturally libidinous temperament may confer a disposition, which habitual indulgence, with the absence of those restraints that modesty, society, and religion impose, may eventually confirm: nay we think it highly probable that many of those conditions of the vagina and uterus which have been adduced with confidence as causes, have really been consequences of the malady.

We now return to Dr. Davis, and to the physical history of nymphomania. Pruritus of the external organs is a well known cause of nymphomania. This pruritus is said to be sometimes occasioned by ascarides in the rectum. The following case would seem to prove that irritating injections into the rectum will occasion nymphomania itself.

*Case* 1. Catherine B., æt. 58, of a sanguineous and highly irascible temperament, became the subject, about the period of the cessation of the menses, of an herpetic eruption, together with a very troublesome pruritus of the external parts of generation. Soon after its first appearance, its symptoms yielded to a regulated regimen and suitable medicines; but in about two years afterwards they again returned. The patient, on that occasion, consulted an herbalist, who engaged to effect a radical cure of her complaint. For that purpose, he made use of injections consisting of certain preparations of drastic plants, such as of the hedge hyssop and asarum. The two first of these enemata produced copious evacuations. That effect was, however, accompanied by an exceedingly troublesome itching of the genitals. The third excited an insatiable desire for sexual intercourse, and was followed by very profuse evacuations, with faintings. A fourth produced another morbid affection: deglutition became impossible, and the approach of fluids seemed to have the effect of closing up the throat, and of exciting a sense of suffocation; the patient, in the mean time, complaining of a burning heat from the throat down to the epigastrium. The horror of liquids became more and more distressing, until at length the very sight of them caused convulsions. During the subsequent night, the patient experienced several paroxysms of phrenitic delirium, and on more than one occasion she manifested a disposition to bite. On the third day she was observed to discharge great quantities of saliva; the pulse was small and intermittent; the extremities became cold, and after a lapse of a few hours she expired.

Certain articles of diet, cantharides, pessaries, are said to have occasioned the disease. Whilst on this part of his subject, Dr. Davis introduces a case communicated to him by Dr. Lucas, of Brecknock, in which that gentleman was obliged to extract from the cavity of the *uterus*, the stock of the spindle of a spinning-wheel. An incision of the os uteri was necessary to complete the extraction of the foreign body. Our readers may call to mind cases in some degree similar. We are not aware, however, of any in which foreign bodies of any magnitude had got into the *uterus*.

Worms in the uterus have been supposed, indeed asserted, to be among the causes productive of nymphomania. Checked perspiration, menstruation, &c. have also been regarded as causes; but it is difficult to conceive that they could prove adequate to the effect. The suppression of the catamenia may perhaps be excepted, for alterations of that function and secretion exercise a most important influence upon the female. A case in point is quoted by Dr. Davis. The nymphomania was periodical, and ceased on the re-establishment of the menstrual flux. In some instances the tendency to the disease would appear to be connate.

*Case.* "A child was addicted even at three years of age to gross personal habits. As she grew up, her disgusting propensities grew with her, and became more and more intolerable. Her parents made an early arrangement for her marriage, and made choice for her of a healthy and robust husband. In due time she became pregnant, and for a short period subsequently was partially relieved from the importunities of her malady, without however experiencing at any time the ordinary amplitude of satisfaction incident to a frequent enjoyment of the privileges of the state of marriage. Her delivery, when her period of gestation arrived, was attended with great difficulty; or rather she died in the midst of the struggles of parturition. On exposure of the genitals after death, the clitoris was observed to be of the size of the male organ. The periods of greatest salacity of the subject of this miserable case had been those of the beginning and end of spring in every year; and at those times there emanated from her person a strong offensive odour similar to that of goats. Her inordinate propensities were represented as having been in some respects hereditary."

Enlargement of the clitoris is a well known accompaniment of nymphomania. In some patients who have died, inflammation and even sphacelation of the vagina and inferior portions of the uterus have been discovered. But these may be effects, as we observed before. In other cases, chronic disease of the uterus and its abdominal appendages has been disclosed. A case of the latter description has lately been published by Dr. Bright, and may be introduced here—it is quoted by Dr. Davis.

"Mrs. M., at the age of 74, became the subject of a most decided and violent nymphomania, with such aberration of mind, and such furious demonstrations of the peculiar turn of her feelings, as rendered it necessary, on several occasions, to subject her to personal restraint. She had been a stout, healthy woman, had been twice married, and was the mother of eight living children. During the continuance of this peculiar derangement, which lasted at intervals till her death, she was two or three times the subject of slight and transient paralysis, principally affecting the organs of speech; and she latterly became somewhat imbecile; and from being a remarkably active woman, became inert and listless; she lived, however, five years from the first attack of her ailments. Sectio cadaveris. The head was not examined; but the only disease discovered in the other cavities was a considerable calculus, apparently of cholesterine in the gall-bladder, and a peculiar and marked disease in the uterus. At the os uteri, and growing from it, was a tumour of a vesicular character, of the size of a large hazel-nut, containing a transparent fluid, and projecting towards the vagina. The cervix uteri itself was much thickened and hard to the touch, and on being cut through, two or three cysts of the size of peas were seen in its substance. About half an inch from the tumour just described, attached to one side of the uterus internally, another similar tumour arose, evidently composed of four or five cysts, parts of which were seen through the membrane which covered the whole. Having divided this tumour by cutting down upon it carefully, a cyst of considerable size was laid open in the body of the tumour, and from the bottom of that arose a globular vesicular body. Still farther along the internal cavity of the uterus might likewise be seen indications, though less obvious, of similar vesicles forming within the substance of the organ."

Two fatal cases are related by Dr. Bright and transcribed by Dr. Davis. The subject of the first was bled thirty times within six days. On dissection the clitoris was found enlarged, as were the ovaries. The fallopian tubes were sufficiently developed to lodge a full-grown pea-shell. In the second case the uterus, ovaries, and fallopian tubes were found in a state of acute inflammation. In another case, the right ovary was found as large as a man's fist, and contained a corresponding quantity of serum.

Dr. Davis enters into the consideration of eroto-mania, which he distinguishes, and very properly, from nympho-mania. In the former there may exist no excessive libidinous propensity; in the latter it is indispensable. A long, but a characteristic case of eroto-mania is related by Dr. Davis; we cannot afford space for it, but many are to be found in the works of Esquirol and others.

It is difficult to say where the passion of love ends and mania begins. A female, for instance, disappointed in an attachment, refuses all intercourse with her family and the world, rejects food and medicine, and in a short time dies. Another commits suicide. A third becomes maniacal. A fourth is attacked with nymphomania. We leave those who can, to decide what are and what are not the legitimate effects of unrequited or unhappy passion, and what the result of disease. Certain it is that a female may die of love, who evinces no other symptom of mania—that erotomania may exist independent of inordinate desires—and that nymphomania is not necessarily attended with any perceptible aberration of intellect. On the other hand, it is equally certain that the barriers between these states are not always evident.

We proceed to a consideration of no mean importance, the treatment of the disease. This is clearly divisible into two portions—the moral and the physical. Dr. Davis divides it into that of the sentiment and that of the sexual passion. We confess that the distinction does not appear so obvious to us as to its author, and we find it difficult to reduce his cases and his doctrines into a very consistent epitome. He commences with the treatment of disappointed love, and as we have no other nor better to offer, we subjoin a sketch of the Doctor's.

He observes that one of the earliest and most prominent effects is a loss of the power of sleeping, and great cerebral excitement. As the lover cannot be procured, a substitute must be discovered, and that is opium. Nothing less than from two to three grains, or an equivalent quantity of the other forms should be prescribed, and a third or a fourth of this should be continued every four or five hours till sleep is procured. In most cases of preternatural cerebral excitement, it should be made a question whether the abstraction of fifteen to thirty ounces of blood should not precede the exhibition of opium. As a good accompaniment to opium, a pretty ample dose of calomel or blue-pill may, in many cases, be added. Such is the Doctor's methodus medendi. We fear that the physician is generally at a discount in these affairs.

Leaving the management of "the sentiment," we come to that of the "passion," in other words, to that of nymphomania. As the causes of this affection are various, the first thing to be done is to discover that of the individual case. Dr. D. observes that, the circumstances that usually present themselves most prominently, are increased constitutional excitement and local phlogosis.

"To meet the important indication here proposed, an ample abstraction of blood will naturally suggest itself to a judicious practitioner as a first and indispensable measure.

If the disease be recent, the first general bleeding should be such as to produce full fainting; and it should be afterwards speedily followed up by the application of leeches to the genitals. When it shall be practicable to convey these auxiliary operators high up into the vagina, and as far as the orifice of the uterus, a number of them not exceeding eight or ten might suffice for one time; see p. 311 and 389: but if that could not be done, and it were only practicable to apply them to the external genital surfaces, the application at least of twenty would be required in order to obtain an equal amount of depletion from the uterine system.

On the removal of the leeches the patient should be placed in a tepid or sub-tepid hip-bath; where she might remain with great advantage for several hours; the temperature of the water to be duly proportioned to that of the central parts of her person and to the excited state of her circulation. This part of the treatment, whilst it should promote a continuous bleeding from the leech wounds, would also most probably be followed by much diminution of excitement of the general vascular system, as also by a corresponding reduction of the morbid fulness and phlogosis of that of the uterus and its appendages."

In addition to these means, it might probably be necessary and useful to apply cold to the head, from which the hair might be shaven. The bowels should be opened by a dose of calomel and jalap, and the remaining indication, on the practitioner's first day of attendance, would be the procuring of sleep by a powerful opiate.

"On the second and some of the succeeding days of the disease it would probably be necessary to have recourse to a repetition of several of the measures now recommended for the first day's adoption. The local bleeding, e. g. might be required to be repeated, as might also the use of the sub-tepid hip-bath.

To ensure a successful application of leeches, whether to the orifice of the uterus, or to the external genitals, it would almost always be necessary to place the patient under so much personal restraint as should prevent the possibility of her changing her position, or making use of her hands, whilst the duty was performing. The use of the strait-waistcoat has also been especially recommended in cases of this kind, to keep the hands from being improperly used in reference to certain propensities and peculiar importunities of the disease. Whilst leeches were being applied, the opportunity should be made available for examining with great accuracy the condition of the parts affected in order to ascertain first the amount and extent of their inflammation; and secondly, the kind, quantity, and if possible the precise source, of any morbid secretions which might be furnished by the phlogosed organs. If immediately, on the falling off or removal of the leeches, it should be inconvenient or otherwise unadvisable to have recourse to a repetition of the sub-tepid bath, as recommended among the items of the first day's practice, a very useful substitute might be found in the injection of full and repeated charges of cold water into the vagina, together with an occasional injection of the same fluid into the rectum. In cases of extreme phlogosis it might be prudent to attempt a gradual reduction of the heat of the parts by using first, tepid water to them, and then successively the same fluid in gradually reduced degrees of temperature. In cases of great offensiveness of the discharges, it might be exceedingly useful to syringe the affected surfaces two or three times daily with a moderately stimulant injection, consisting of equal quantities of decoction of bark, camphor mixture, and port wine. Under the more ordinary forms of inflammation of the same tissues, a solution of nitrate of silver in water, in the proportion of eight or ten grains of the salt to a pint of water, would furnish a better application. A reduction of the temperature of the parts within the pelvis has sometimes been attempted by a constant wearing and frequent changes of napkins charged with cold and evaporating fluids. It would appear doubtful whether and for how long a time the use of these and similar applications should be persisted in subsequently to the subsidence of the disease into a chronic form. The author knows but of one case which he can appeal to in illustration of the point in question."

Dr. Davis remarks that, in the first stage of nymphomania, vigorous and appropriate treatment may, with good reason, be thought likely to remove the disease, unless it depend on structural alterations. In the second stage a cure is less probable, but not hopeless. In the third, that of absolute fatuity, the chance of restoration is small indeed. Some further incidental remarks on treatment made by Dr. Davis, may be comprised in a few words—reduction of temperature about the central portion of the body—personal restraint by the strait-waistcoat or a flannel bandage constantly moistened—the face to be dashed with cold water on the manifestation of indecency—cool and country air—light and suitable clothing, with warmth to the feet—vegetable and unirritating diet. On the moral treatment we need not descant. It must be suited to the individual case, and regulated by the judgment of the family and the medical attendant.

---

### XV. ON SOME POINTS CONNECTED WITH THE ANATOMY AND SURGERY OF INGUINAL AND FEMORAL HERNIÆ, &c. &c. By G. J. GUTHRIE, Esq. F.R.S. &c. &c.

WE need scarcely say that whatever falls from Mr. Guthrie is valuable and interesting. His great experience, his ingenuity, and his talents, combine to recommend any work of his to the notice of the public. We have seized the earliest opportunity of laying the substance of the present pamphlet before our readers. The able author informs us, in a short preface, that it was written nearly two years ago, and that he has deferred its publication to the present time, in the hope that the observations contained in it, might form a part of the first volume of the Transactions of the Royal College of Surgeons in London—a hope that has been disappointed.

Contrary to our usual custom, this notice will not be analytical. The chief object of the author is to offer a more correct description of the anatomy of inguinal hernia. Such descriptive writing cannot be analyzed. We must content ourselves with extracting such passages as will give our readers an accurate notion of Mr. Guthrie's views, connecting those passages as well and as clearly as we can. We may remark in limine, that the substance of the present paper was contained in the lectures delivered by Mr. Guthrie, in the Theatre of the Royal College of Surgeons, in February, 1831.

Mr. Guthrie commences by mentioning what always appeared to him a difficulty in comprehending the cause of stricture in some cases of inguinal hernia. If it were commonly admitted that the inner opening through which an inguinal hernia proceeds, were surrounded by muscular fibres, there would then be no obstacle in accounting for the circumstances connected with the strangulation of the hernia. But such muscularity is not commonly described nor allowed, and Mr. Guthrie has long conceived that it is impossible, or nearly so, to explain the facts without it. He goes on to observe,—

" Before I was enabled to demonstrate the muscular structure of these parts, I had had the opportunity of examining the bodies of two persons who had died from strangulated herniæ, in both of whom the stricture on the intestine had been so great, that a common silver probe could not be easily passed in the canal of the gut. The last case was that of a person who had been operated upon, and died shortly afterwards; the intestine had been returned into the cavity of the abdomen, and was found lying behind the inner ring, with a narrow but deep indentation around it, marking the place at which the stricture had existed, and through which a probe could only be passed by dilating the contraction. I showed the preparation at my lecture, and declared, what I believe to be true, that this could only have taken place from some direct muscular pressure from without, and not from any congestion or dilatation from within or below the stricture. I also acknowledged that I could not shew, by dissection, in what manner this contraction had taken place, or by what parts it was effected. I have always, however, continued to impress upon the minds of the gentlemen attending my lectures for the last ten years, that the principal cause of strangulation in recent hernia was a contraction of the superior or internal opening of the inguinal canal, the cause and nature of which I could not satisfactorily explain, that it was, therefore, a point in anatomy deserving investigation, for a discrepancy of this kind could not exist in nature; and that there must be something defective in our knowledge of the subject."

To shew that he is not combating a shadow, Mr. Guthrie cites the opinions of various anatomical and surgical authors of eminence, on the nature of the inguinal passage and openings. These we need not quote; suffice it to say, that none describe muscular fibres, so disposed as, in any part, to encircle a hernial protrusion and contribute to its strangulation. We pass therefore to Mr. Guthrie's correction of former writers, premising M. Cloquet's description of the fascia transversalis, which is indispensable to the right understanding of Mr. Guthrie's.

M. Cloquet states, that it is an aponeurosis, the thickness of which varies: it springs from the posterior edge of the crural arch (Poupart's ligament), from the aponeurosis of the iliac muscle, from the external border of the rectus muscle, and is continued upwards with the cellular tissue on the internal surface of the abdominal muscles: below and towards the middle of the crural arch this aponeurosis gives rise to a membranous canal, commencing by a wide opening directed backwards and outwards, the internal edge of which is thicker than the outer. This canal descends around the spermatic vessels, forming their proper sheath. The fascia transversalis supports the peritoneum, which is behind it, the epigastric artery passing between them. Before, it corresponds to the transversalis muscle, with the aponeurosis of which it is often so closely united that it can only be distinguished from it by the different direction of its fibres.

Again M. Cloquet remarks that, very often the fascia transversalis is evidently formed of two aponeurotic layers, which are united on a level with the top of the crural arch. Of these the anterior comes from the arch itself (Poupart's ligament), the posterior being only a continuation of the fascia iliaca, which quits the iliac muscle to ascend upon the anterior wall of the abdomen. These two layers thus re-united proceed back to back between the transversalis muscle and the peritoneum. It is easy to separate them on the outside of the superior opening of the inguinal canal; but on the inside and around it they are intimately united. When this formation is met with, the posterior layer passes usually behind the rectus muscle in its way to the linea alba, whilst the anterior one is continuous with the edge of the tendon of the rectus. The epigastric artery is sometimes posterior, sometimes anterior, and sometimes even between these two layers.

Mr. Guthrie thinks that this, which Cloquet considers an accidental occurrence, is really the normal condition, and that if the fascia transversalis be said to be composed of two layers, the anterior fibrous, the posterior cellular, much confusion will be avoided. Mr. G. goes on to say that, if the student is taught to consider the fascia transversalis as a sheet of condensed cellular membrane, divisible in some parts into two layers, passing upwards from Poupart's ligament to fortify the peritoneum, he will readily understand it; and if he is shewn that at a certain spot it becomes much thinner, and allows the spermatic cord to pass through, he cannot fall into any misapprehension. This part is not, however, an opening; it is merely the thin portion of the fascia which, as the testis escaped from the abdomen, was carried forward by that gland, and is now seen attaching itself to the spermatic cord. If this cord be drawn down, and an incision be made around it, close to where it is attached to the peritoneum, a sort of ring is formed, and if the finger be introduced, the thin part can be stretched or torn, until the firm internal edge of the denser anterior layer of fascia transversalis can be distinctly seen, having the epigastric artery a little to its inner side. The outer side of the ring is not so well marked, and the hole thus made by the finger is usually so large, and its outer edge so weak, as to occasion little fear of any great constriction being made by it on any portion of the contents of the abdomen which may be protruded through it. It is therefore not the part which constitutes the stricture at what is called the inner ring.

The following quotation will, we believe, give the reader an idea of Mr. Guthrie's views of the anatomy of the transversalis fascia and muscle. With it we must close this notice, recommending all to peruse the original paper. In addition to the descriptive portion, there are many practical hints of much value. We rejoice to see that Mr. Guthrie promises the public some farther contributions on surgical subjects. We trust that the promise will be kept.

"When the peritoneum is carefully removed from the inside of the wall of the abdomen, by tearing through the cellular membrane which attaches the one to the other; the fibrous or anterior layer of fascia transversalis is not the part next brought into view, but a distinct layer of cellular structure resembling fascia, although oftentimes loaded with fat, which can be readily dissected off in a complete sheet, carrying with it the epigastric vessels which adhere rather to it than to the fibrous texture in front. This cellular layer I take to be the same thing as the posterior layer of Cloquet; but whether it is or not, it passes behind the rectus to meet its fellow from the opposite side, covers the iliac vessels below and passes under Poupart's ligament, forming their cellular and adipose sheath and the septum which passes between them. When this cellular layer is turned down, (as in Plate III. fig. 1.) the fibrous fascia transversalis is brought into view. If an attempt is made to turn the latter down from the transversalis muscle two inches above Poupart's ligament, it is often found to adhere very firmly to it, and to its aponeurosis. When muscular at this part, many of the fibres seem to be implanted on it, although both muscle and aponeurosis are sometimes wanting, in which case only can the spermatic cord lie upon the fascia transversalis. When the tendons of the internal oblique and transversalis muscles are complete and well marked, and they and the fascia transversalis are traced inwards to the rectus muscle, the two tendons are seen to pass in front of it very distinctly; the fibrous layer of the fascia transversalis on the contrary divides, the anterior and thickest part is attached to the anterior edge of the rectus; the other, which is very thin, passes behind the rectus to meet its fellow from the opposite side; but at the lower part close to the pubes, a portion of the fascia transversalis becomes very strong, and resembles, more than any thing else, a round white tendon going to be inserted into the pubes near its symphysis, and behind the rectus. The interspace formed by the recedence of the two insertions is very distinct, the rectus filling it up. The fascia transversalis passing externally from this sort of tendinous insertion, is attached to the inside of Gimbernat's ligament, of which it forms the internal layer, and is then continuous with the pelvic fascia. Passing from the upper edge of Gimbernat's ligament, or the third insertion of the external oblique muscle, to the inner edge of Poupart's ligament, or the second insertion of the same muscle, the fascia transversalis seems to adhere so strongly as to appear to be a reflection upwards from it, which is the view taken of it by the French anatomists: but if care be taken in making the dissection, it can with some difficulty be separated from it, and be shown to pass under the ligament to form the septum crurale, and the anterior part of the sheath of the femoral vessels. Exterior to the femoral artery the fascia transversalis is firmly attached to Poupart's ligament, and is continuous with the iliac fascia.

The transversalis muscle lies immediately upon the fascia transversalis. Its inferior edge is said to pass over the spermatic cord at the inner or superior opening of the ring, in order to form, with the internal oblique, the sheath of the rectus. This I believe to be in many instances an incorrect description. In the demonstration of these parts in the Theatre of the Royal College of Surgeons, I had the opportunity of showing (see Plate I.) the transversalis muscle advancing fleshy or muscular, until it reached the spermatic cord; a portion of it then took the usual course above and over it, whilst another portion passed below it, the terminating muscular fibres of which were inserted along the inner edge of Poupart's ligament up to the pubes. The muscularity of this insertion was admitted by the various teachers of anatomy, and other competent judges who were present. The lower part of the abdomen was thus shown to be defended by a layer of muscular and tendinous fibres, lying upon the fibrous layer of the fascia transversalis; and the spermatic cord passed, not, as it is usually stated, under the inferior edge of the transversalis muscle, but through a split in it originally formed for the purpose of giving passage to the testis. This split or opening was rounded on its under part where the spermatic cord rested upon it, and formed a small opening essentially muscular in every direction, and much less in size than that which is described as the opening of the fascia transversalis, which adheres to the internal surface of the muscle.

It is this part therefore, and not the fascia transversalis alone, which constitutes the inner or superior opening of the inguinal canal for all surgical purposes. The transversalis muscle does not, however, in the generality of instances, send its inferior portion forwards and beneath the spermatic cord in so marked a manner. This part of the muscle more frequently becomes tendinous and aponeurotic; but its fibres, although tendinous, are distinctly marked, running transversely in continuity with the fleshy fibres of the muscle, and are inseparably united to Poupart's ligament. In some instances the muscular fibres of the transversalis do not take so oblique a direction from without inwards and downwards, but crossing more horizontally, send down a narrow tendon, on forming with the internal oblique the sheath of the rectus, which descends almost perpendicularly for some distance, to be inserted into the tuberosity or spine of the os pubis. The inferior or aponeurotic part of the transversalis may be equally present, forming the inferior edge of the inner opening of the inguinal canal; but this formation cannot take place if the spermatic cord passes immediately over Poupart's ligament, in which case, this ligament forms the under part of the inner opening of the inguinal canal, the lower edge of the transversalis muscle the upper, and the fascia transversalis the sides. The epigastric artery runs within a few lines of distance from the internal edge of this part or opening; and between this vessel, on the outside, the edge of the rectus on the other, and Poupart's ligament below as the base, the triangular space of Hesselbach is formed, through which that sort of hernia takes place, which is called *internal* by him, but *direct* by Sir Astley Cooper, to distinguish it from the more common one which, passing through the inner or superior opening of the ring, is called *external* by Hesselbach, and *oblique inguinal* hernia by Sir Astley Cooper.

When the transversalis muscle is inserted broadly into Poupart's ligament by its superior fibres only, the anterior ones pass on to form the sheath of the rectus, and to be inserted into the tuberosity of the os pubis; but a layer of fibres internal to these (the folded fibres of Sir Astley Cooper,) are implanted on the fibrous external layer of the fascia transversalis, and curve downwards to be inserted into Poupart's ligament, or proceed, according to Breschet, to form what he calls the pretended ligament of Gimbernat, and which he will not admit to be a third insertion of the external oblique muscle. These fibres are depicted by Cloquet (Plate I. fig. 3) as belonging to the fascia transversalis, and by Breschet (Plate III. fig. I.), in his *Considérations et Observations Anatomiques et Pathologiques sur la Hernie Femorale,* Paris 1819, as essentially going to form the internal layer of Gimbernat's ligament, of which the fascia lata of the thigh supplies the outer. They are very distictly shown in Plate III. fig. 1., and certain fibres running in a less curved and more vertical direction, belonging to the anterior or fibrous layer of the fascia transversalis, are equally well marked. These fortify this part in an especial manner, and something like the way in which the outer angle of the external ring is strengthened by fibres crossing in a similar manner."

### XVI. CHARACTER OF THE ESQUIMAUX.

THE Phrenological Journal for September of the present year contains an interesting article on the peculiarities of this singular people, if people they deserve to be considered. The letterpress is accompanied with drawings of the skull of an Esquimaux, and of a Papuan, or inhabitant of New Guinea. In a table are contained the phrenological dimensions of twelve Esquimaux skulls, the originals, or the casts of which, are at present in the Society's collection. The table is too bulky for our columns, but we select a portion of it, representing the average size of the various organs in these hyperborean savages. The dimensions are as follow :—

Amativeness, $17\frac{1}{4}$—Philoprogenitiveness, $18\frac{5}{6}$—Concentrativeness, $15\frac{3}{4}$—Adhesiveness, $14\frac{1}{6}$—Combativeness, 16—Destructiveness, $15\frac{1}{2}$—Secretiveness, $15\frac{1}{3}$—Acquisitiveness, $13\frac{1}{4}$—Constructiveness, 14—Self-esteem, $16\frac{1}{2}$—Love of Approbation, $15\frac{1}{3}$—Cautiousness, 15—Benevolence, $13\frac{1}{6}$—Veneration, 16—

Firmness, $15\frac{1}{3}$—Conscientiousness, $9\frac{5}{6}$—Hope, $10\frac{1}{2}$—Wonder, $10\frac{5}{6}$—Ideality, $10_{6}$ Wit, $10\frac{1}{3}$—Imitation, $11\frac{2}{3}$—Individuality, $12\frac{2}{3}$—Form, $9_{6}$—Size, 10—Weight, $9\frac{1}{4}$—Colouring, $8\frac{1}{4}$—Locality, 11—Number, $8\frac{2}{3}$—Order, $8\frac{1}{4}$—Eventuality, $10\frac{3}{4}$—Time, 9—Tune, $9\frac{1}{2}$—Comparison, $11\frac{1}{4}$—Causality, $10\frac{1}{2}$.

So much for the cerebral development. A brief glance at the character of these barbarians may not be totally uninteresting to the medical reader. Their mental peculiarities may be speedily summed up, and perhaps the following enumeration is a fair approximation to the truth:—extreme affection for their children—a strong venereal appetite, rendering prostitution universal, open, and unblushing—fearlessness of danger, and little distrust of each other or of strangers—improvidence in providing for future want, and reckless enjoyment of the present plenty—courage in the chase of the Polar bear, and little disposition to mutual contests—hospitality and meanness, conjoined with a disposition to dishonesty so notorious as to lend a hue to the Esquimaux race—and that remarkable contempt for Europeans and their arts, for civilization and its benefits, which has often stamped the most debased, most impotent, and most ignorant of mankind.

The latter feature has frequently exercised the reflection of the philosopher and the satire of the wit. The happy tale of the traveller in the Alps, so aptly conceived and so felicitously told by Goldsmith, is probably familiar to the majority of our readers. If the fictitious congregation of that story could burst into laughter at the absence of a goitre, it did no more than the Esquimaux or the American have done upon similar occasions.

"Superior," says Parry, "as our arts, contrivances, and materials must unquestionably have appeared to them, and eager as they were to profit by this superiority, yet, contradictory as it may seem, they certainly looked upon us in many respects with profound contempt; maintaining that idea of self-sufficiency which has induced them, in common with the rest of their nation, to call themselves, by way of distinction, *Innuee*, or mankind. One day, for instance, in securing some of the geer of a sledge, Okotook broke a part of it composed of a piece of our white line, and I shall never forget the contemptuous sneer with which he muttered in soliloquy the word 'Kabloona!' (European), in token of the inferiority of our materials to his own. It is happy, perhaps, when people possessing so few of the good things of this life can be thus contented with the little allotted to them."

A Tartar robber, seated, as Voltaire has happily expressed it, in a miserable hut, proclaims each day before he takes his meal of milk, that the princes of the earth have his sovereign permission to go to dinner. The meanest of mankind have ever been inflated with the wildest conceit, and Goldsmith has said well, that "the most ignorant nations have always been found to think most highly of themselves." The same extraordinary phenomenon is observed, and that not rarely, in the walks of civilized life. The ignorant are frequently most arrogant, most positive, most fraught with the notion of their own importance, and the littleness of all without their magic circle of self.

The moralist may grieve and the man of society may laugh at this strange perversity of human character; it is the part of the philosopher to analyse it and explain it. Unfortunately the investigation of the mind has been hitherto withdrawn from those best acquainted with its ordinary workings, and the cumbrous machinery of metaphysics has surrounded and protected mental philosophy, from the mass of the medical profession.

"The high estimate which savages form of their own importance, is undoubtedly to be ascribed to the activity of Self-Esteem, a faculty which, when 'powerful and ill-regulated,'—as it generally is, among uneducated and ignorant individuals,—'fills the mind with unbounded sentiments of self-excellence, without reference to merit.' The absence of that extent of misery which civilized nations are apt to look for in circumstances so wretched as those above described, may, we think, be accounted for, by attending to the constitutional qualities which savages seem to possess. We have already seen that the Esquimaux are distinguished not only by moderate organs of the intellectual faculties and Ideality, but also by a lymphatic temperament, indicative of little activity of the nervous system, including the brain; and it is highly probable that the same constitution will be found to prevail among such tribes as the inhabitants of Terra del Fuego and New Holland. Now, as desire is the effect of, and in proportion to, the activity of the faculties, these savages, possessing feeble and sluggish intellectual powers and Ideality, have few or no desires thence originating; their moral and intellectual faculties are, as it were, half asleep; their sensations are blunt; and they suffer little uneasiness from exposure to influences which, in the case of men with active nervous systems, would be productive of acute suffering. As their means of gratifying desires are scanty, so those desires are few. 'Happiness,' in the words of Spurzheim, 'depends on the gratification of active faculties, and unhappiness on their non-satisfaction.' 'He who has many faculties active which he can satisfy, is more happy than he who has no desire whatever; it is, however, better to be without desire than to possess very active faculties, with no means of ministering to their cravings.'

The happiness of savages seems to consist more in the absence of disagreeable sensations, than in the experience of a variety of pleasurable emotions. Nearly the whole of their enjoyments have reference to their animal nature alone; their minds have no longing after unattainable felicity; and when the few desires which they possess are satisfied, they experience perfect contentment. Civilized man has many desires, to the cravings of which he frequently cannot administer; and, in consequsnce, he suffers misery.

If he could satisfy them all, he would have a greater amount of happiness than the savage at the limit of *his* felicity; for enjoyment is great in proportion to the number of satisfied desires. We have frequently expressed a conviction that the misery which at present scourges civilized nations is in a great measure the result of ignorance and folly; and that, by attending to the conditions prescribed by the Creator for the attainment of happiness, the amount of human suffering may be incredibly diminished. By the extension of knowledge, men will be enabled to regulate their desires to a much greater extent than is now practicable, and at the same time to provide more complete gratification to such desires as they possess."

If we look at this question without a particular reference to phrenology, we shall find little difficulty in explaining much of the vanity and happiness of barbarian tribes. Wants and wishes are chiefly the result of circumstances, and their origin, as their gratification, depends on the condition of the external world around us. There are some wants which are instinctive, and must be gratified. Such are those of hunger and of thirst, &c. animal necessities felt by the savage as the sage. But beyond a certain barrier all is artificial, the creature of the circumstances in which the individual is placed.

In civilized life, the country maiden, accustomed to see others situated like herself, has little desire, and scarcely a conception, of more than the homely fare and the rustic garb which her family and her neighbours are possessed of. But, introduced to the town, new objects present themselves and new desires arise. We may readily account for the content of savages under seeming privations, by reflecting that, to them, they are not privations, but really the enjoyment of all that they ever expected to enjoy. We may bring the case home to ourselves, by supposing a race infinitely more civilized, or more favoured by nature, than we are, looking down on our acquisitions and our happiness with equal surprise with that which we exhibit towards the Esquimaux.

It is not so easy to explain the pride of barbarians, and the obvious inconsistency of their contempt for civilization, and eager endeavours to acquire its gifts. It is probable that this feeling does not long subsist, and that acquaintance with the civilized ends is a more rational appreciation of their superiority. Such has been the case with the American Indians. We may partly account for this feeling of vanity, by reflecting that savages are usually much courted and flattered in the first instance. The Esquimaux sees the white man come to his inhospitable clime, and he naturally supposes that some advantage possessed by it has brought the stranger to his shores. He sees little more than a handful of men deserting their homes, wherever they may be, to visit him, courting his friendship, and purchasing his comforts. It is not a matter of astonishment, that a hasty intercourse should impress him with satisfaction at his own condition, and pride at himself and his country being the object of attention.

---

### XVII. Fatty Discharge from the Bowels.

A case of this sort has been related by Mr. Eastcott, Surgeon to the St. Pancras Parochial Infirmary.

The patient, a woman, æt. 48, was admitted into the Infirmary Feb. 5th, 1833. She had been a hard drinker, and was of very irregular habits. She had long been ill, and appeared to be suffering from organic disease of the liver. She had a greenish cast of countenance and was emaciated; complained of considerable pain in the right side and epigastrium; was subject occasionally to severe pain in the bowels; but what was more remarkable, she passed with her stools, which were very bad, and shewed a great deficiency of bile, a discharge resembling tallow, or spermaceti, or like a mixture of both, which floated on the surface of the urine in the vessel in considerable quantities. Was treated with hyd. c̄ creta in small quantities, leeches, and blisters, and supported with mild nourishment. She died Feb. 17th.

*Dissection.* "Lungs tolerably healthy, but effusion of serum into the cavities of the pleura—altogether about three pints. Liver soft, and somewhat large, of a pale yellow colour (the colour of Flanders brick), and its surface spotted with petechiæ. Gall-bladder transparent, not in the least degree tinged with bile, but resembled a particularly thin and well-washed bowel; it contained about two ounces of a thin fluid, more like bilious urine than bile. The colon very contracted—a mere rope; on its mucous membrane, which was very pale, and which was examined throughout its whole course, no trace of inflammation presented itself; the contents, except near the rectum, where there was some appearance of fæcal matter, was a whitish secretion resembling blanc-mange, and in other parts of it like thick rice gruel. I saw nothing like the matter which had been previously discharged per anum. An old adhesion existed between the omentum and gall-bladder. The pancreas was hardened, and changed in structure, and the pancreatic duct, which was enlarged, contained a number of solid chalky concretions as hard as bone, some about the size of peas, some larger, and resembling in roughness of their surface the 'mulberry calculus' of the bladder. The muscles were attenuated and pale; the cellular substance, particularly about the abdomen, anasarcous; and about two quarts of serous fluid were contained in the abdominal cavity."—*Med. Gaz. April.*

---

### XVIII. Sarsaparilla and the Smilax Aspera.

Dr. Ashburner and Mr. Belinaye have communicated some observations on this subject to the Medical Gazette.* As we have seen a little of the effects of sarsaparilla, and something of those of the smilax aspera, we will notice the more prominent points in the papers of these gentlemen. And first of the physician.

Dr. Ashburner introduced the smilax aspera to the notice of the profession, in the number of the Medical and Physical Journal for March, 1831. He regrets that he has seen no published account of experiments with the drug since that period. The Doctor's additional experience, or rather the additional information which he communicates, appears to amount to this:—

1mo. A note from the Doctor's friend's friend, R—, "the surgeon of Tellicherry," to the following effect. "I have been putting smilax to another

---

* June, 1833.

use. I have had in the hospital three severe cases of venereal. I put them under a course of it. One took it macerated in hot lime-water; another steeped in cold lime-water; and the third in boiling water. All rapidly improved. The ulcers healed beautifully; and one of the patients, who came into the hospital an emaciated, poor, thin, dying devil, soon, under this medicine, became plump and fat." In addition to this, Dr. Ashburner assures the Editor that it has been his lot to observe plumpness and fatness, succeeding to a cachectic condition of body, under the use of preparations of smilax aspera, as well as of smilax sarsaparilla.

2do. After indulging in some reflections on the action of sarsaparilla, and advocating its utility in cachexia and cacocachymia, Dr. Ashburner makes these concluding observations on the smilax aspera.

"In most cases, the use of smilax aspera, like that of sarsaparilla, has been attended with a beneficial change in the condition of the patient, plumpness, clearness and strength, succeeding to emaciation, muddiness, and debility. To insure brevity, I forbear to quote cases in support of my positions, trusting that in time the experience of others will bear out the accuracy of the present report. My own mode of administering this medicine has been that which I originally employed—a pint of the decoction, or of the infusion, in the day. The decoction has been made by boiling a pint and a half of water upon two ounces of the root, one drachm of the extract of liquorice, and half a drachm of the subcarbonate of soda, until the fluid is simmered to a pint. The infusion has been made by steeping two ounces of smilax aspera in a pint of boiling water, or in a pint of lime-water, for twelve hours; straining, and adding to the strained liquor two ounces of the syrup of smilax aspera. This latter form of exhibition is convenient, and the flavour is very agreeable."

Such are the recommendations of the smilax aspera offered by Dr. Ashburner. The remainder are presented to us by Mr. Belinaye, a gentleman, who, acknowledging the Doctor's claim to the credit of bringing the remedy forward, congratulates himself on his having been one of those who have prescribed it most largely. Mr. Belinaye's statements are capable of being contained in a nutshell.

"Two years ago I happened to be called to attend upon a young nobleman, who, after a long course of dissipation, caught the venereal disease. Having taken large doses of mercury, he had to travel home in a great hurry several hundred miles, without this remedy being cleared away, or the disease being perfectly cured. In his peculiar weak state I thought sarsaparilla the best remedy he could use, and I prescribed it. Independently of my apprehension that common sarsaparilla may be a remedy 'qui amuse pendant que la nature guerit,' it is very subject to lay heavy upon the stomach, and to produce indigestion. In the above case the patient could bear it neither in its combination with the alkalies, nor with the mineral acids. Under these circumstances, the 'smilax aspera' happened to come under my notice, and I prescribed it to the complete restoration of the patient's health, who got remarkably fat and strong upon it, and has remained so for the last twenty months. From that time forwards I began to prescribe it frequently. In delicate persons I administered it in combination with one-eighth of a grain, more or less, of the oxymuriate of mercury, every night, and with great success in the cure of syphilis, and of its bastard forms.

The complaint, however, in which I have administered this new remedy most abundantly and successfully, has been gonorrhœa. If it be remembered how difficult gonorrhœa is to treat; that if energetic remedies be administered at first, such as cubebs, copaiva, and injections, the most distressing symptoms in the bladder, groin, &c. may show themselves; or if, on the other hand, gentle remedies be used, the disease frequently degenerates into interminable gleets;—if these circumstances be borne in mind, I think that the usefulness of any new remedy, capable of exerting a certain degree of positive effect, will be easily admitted. The following is the form in which I have prescribed the smilax aspera with efficacy in gonorrhœa, nearly exclusively from the beginning to the end of the malady :—

℞. Liquoris Potassæ ♏xxx. ad ʒj.; Aq. Flor. Aurantii ℥j.; Syrupi Smilacis Aspera ℥v. M. Sumat cochl. ij. ampla ter quaterve in die e Cyatho Decocti hordei magno."

The whole of the question amounts to this. The smilax aspera is more cheap than sarsaparilla—is it as efficacious? It is a point of relative value. The public would suppose, that, to establish the equal efficacy of the two, or the superior merit of one, comparative facts would be related. The advocates of the smilax aspera might fairly be called upon to demonstrate its powers in one of the following modes—either to select cases in which sarsaparilla was peculiarly beneficial, and then by exhibiting the smilax aspera to shew its equally good effects;—or, to going farther than this, to bring forward instances in which sarsaparilla failed, and the smilax aspera succeeded. The first series of experiments would go towards establishing the equality of the two—the last would display the superiority of the latter.

But this is not done by either of the advocates of the smilax aspera, and, what is singular, their statements would appear diametrically opposed on a very important point. The tenor of Dr. Ashburner's paper is manifestly in favour of the powers of sarsaparilla. He admits them, extols them, and would seem to lament that able provincial practitioners should doubt them. Mr. Belinaye, upon the other hand, is one of those sceptics, whose state of mind is virtually deplored by Dr. Ashburner. He expresses an apprehension that "common sarsaparilla may be a remedy, *qui amuse pendant que la nature guerit.*" It is strange, that two observers who start with such different views should arrive at the same conclusions, and it does seem impossible to us to reconcile the evidence of men who have not only no common standard, but whose standard is at opposite extremities of the scale.

We are therefore of opinion that the papers of Dr. Ashburner and Mr. Belinaye afford the public no opportunity of determining the relative virtues of sarsaparilla and the smilax aspera. They merely shew that this remedy has been employed by these gentlemen in certain diseases, with certain effects.

We may, probably, be permitted to take a slight glance at the results.

Dr. Ashburner employs the smilax aspera in the cases in which he would employ sarsaparilla, chiefly those of a cachectic character. The essential consequence observed by him is, improvement of the general health, displayed by the amelioration of the complexion and the acquisition of fat.

Dr. Ashburner's friend, the Indian surgeon, has given the smilax aspera in "three severe cases of venereal." The ulcers "healed beautifully" under the treatment, and one of the patients, who was thin, got fat.

Mr. Belinaye prescribes it, in combination with the oxymuriate, for the cure of syphilis and "its bastard forms," in delicate individuals. But his chief employment of the medicine, and its chief success, is in the treatment of gonorrhœa.

So far as our own experience goes, Dr. Ashburner's view of the probable value of the remedy is most consistent with the truth. Whatever may be the immediate action of sarsaparilla, its ultimate effect is to amend the general health, and the cases most adapted to it, and most improved by it, are those in which the general health is defective. But sarsaparilla is certainly not a specific for syphilis, and we therefore attach very little importance to the statement of the Indian surgeon, that three cases of the venereal were cured by the smilax aspera. What confirms us in this scepticism is the consideration, that "the venereal" is a term sufficiently comprehensive to include many cases not syphilitic, and that even if the sores of syphilis be healed, the malady is not of necessity destroyed.

When Mr. Belinaye treats syphilis in delicate persons, and bastard syphilis too, by the smilax aspera combined with the oxymuriate, it is difficult to say what portion of the result is owing to the one and what to the other. We are sure that syphilis in delicate persons is scarcely more curable by sarsaparilla only, than is syphilis in persons of stronger constitutions, and probably the combination of the oxymuriate with bark, or with other tonics would be quite as salutary as its union with the smilax. It is difficult to pronounce what "the bastard forms" of syphilis are, and the expression is so vague that the treatment can neither be a subject of imitation nor avoidance.

We confess that we feel a little surprise at the reputed efficacy of the smilax aspera in gonorrhœa. We should have felt more satisfaction if particular cases of the disease had been related. We should then have been presented with some data, for calculating the probable amount of the value of the remedy. We confess that a glance at the prescription inclines us to attribute more to the alkali, the syrup, and the barley-water, than to the native virtues of the smilax aspera.

We have hitherto refrained from expressing an opinion on the relative efficacy of sarsaparilla and its rival. Yet our impression is, that the former is greatly preferable. We have tried the smilax aspera, and have seen it tried, with little or with no success, when the substitution of the compound decoction of sarsaparilla was productive of marked advantage. On the other hand, we have heard of cases, in which the smilax aspera was more beneficial than the sarsaparilla, and perhaps there are circumstances, with which we are unacquainted, that may render the former occasionally more beneficial than the latter.

---

### XIX. Dislocation of the Head of the Femur on the Os Pubis, with Fracture of the Femur.

The following method of reduction was successfully adopted in a case of this description by Mr. John Bloxham, an intelligent surgeon of Newport, in the Isle of Wight. We should premise that no attempt at reduction was made for seven or eight days after the injury, short splints, with evaporating lotions, &c. being applied in the interim. At the end of the period we have mentioned Mr. Bloxham proceeded thus.

"The patient was laid on his back on the bed, and kept in that position by means of a sheet passed across the pelvis, and fastened to the bedstead; another sheet was also passed over the left groin, and secured in a similar manner. The dislocated and fractured limb was then enclosed in splints, one of which extended up the back of the thigh as far as the tuberosity of the ischium. Pulleys, which were secured to a staple in the ceiling, placed at the distance of a foot to the right of a point vertical to the patient's navel, were then attached to a bandage fastened round the splints, as high up as possible.

The foot was raised with the knee extended, so as to bring the limb nearly to a right angle with the line of the tackle, when, by drawing gradually on the cord, in the course of about ten or fifteen minutes the head of the bone was rendered moveable, and was brought considerably more forward. I then began to press on the head of the bone, so as to push it downwards, whilst the pulleys held it partially disengaged from the pelvis. In a few minutes the head of the bone passed over the ridge of the os pubis; and I then directed the foot to be raised a little higher, which, by putting the glutei muscles more upon the stretch, was calculated to render them more efficient in drawing the bone into its proper place. By this manœuvre the head of the bone was drawn backwards; and on the foot being more elevated, and the cord slackened, it continued to recede from my fingers till the trochanter major made its appearance in the natural situation, and the reduction was found to be perfectly complete.

Lest the head of the bone should slip backwards on the dorsum ilii, I directed an assistant to apply firm pressure during the latter part of the process, above and behind the acetabulum.

The apparatus was then removed, the thigh bound up in short splints, and the patient laid upon a double inclined plane. No symptoms of inflammation appeared afterwards about the joint. Passive motion was employed at the end of a week, and occasionally repeated during the whole reparatory process." *Med. Gaz. Aug.* 24, 1833.

